# The joint effect of triglyceride-glucose index and C-reactive protein levels on the risk of chronic obstructive pulmonary disease: a prospective cohort study

**DOI:** 10.1186/s12944-025-02732-1

**Published:** 2025-10-06

**Authors:** Jialiu He, Mengxia Li, Pengfei Luo, Zheng Zhu, Jian Su, Ran Tao, Jinyi Zhou, Ming Wu, Xikang Fan

**Affiliations:** 1 Department of Epidemiology and Health Statistics, Southeast University, 87 Dingjiaqiao Road, Nanjing, 210009 China; 2 Jiangsu Provincial Center for Disease Control and Prevention, 172 Jiangsu Road, Nanjing, 210009 China; 3 Department of Epidemiology, School of Public Health, Nanjing Medical University, Nanjing, 211166 China

**Keywords:** Triglyceride-glucose index, C-creative protein, Chronic obstructive pulmonary disease, Joint effect

## Abstract

**Background:**

The triglyceride-glucose index (TyG) and C-reactive protein (CRP) are key biomarkers on clinical diagnosis, each related to lung dysfunction. However, the relationship of both indexes with the risk of chronic obstructive pulmonary disease (COPD) is still unclear. This study purposes to focus on the individual and joint associations of TyG and CRP levels with COPD risk.

**Methods:**

This cohort study utilized baseline TyG and CRP data from the UK Biobank. Hazard ratios (HRs) and 95% confidence intervals (CIs) for COPD risk associated with TyG and CRP levels were calculated through Cox regression models. Receiver operating characteristic (ROC) curves were conducted to determine the optimal cut-off values for TyG and CRP, which were combined into a joint variable. Kaplan-Meier (KM) method was utilized to analyze cumulative hazard, while joint analysis was employed for evaluating the joint risk. Stratified and sensitivity analyses were also performed to assess the associations within subgroups, and mediation effect of TyG on COPD risk via CRP levels was assessed.

**Results:**

This study enrolled 385,523 individuals, with 10,515 COPD cases were recorded in follow-up. Compared to the lowest quintile, individuals with higher TyG and CRP had increased risk of COPD (all HRs > 1.00). The optimal cut-off values of TyG and CRP were 7.14 and 1.88 mg/L, and we found that the simultaneous elevation of both TyG and CRP significantly increased the risk of COPD. Moreover, the joint effect was stronger in participants younger than 60 years old, males, smokers or passive smokers, those with body mass index (BMI) < 25.0 kg/m^2^, and those without baseline diabetes, asthma, or a family history of respiratory diseases (*P*_for interaction_ < 0.05). Moreover, the effect of TyG on COPD was significantly mediated by CRP, explaining almost 15.6% of this influence.

**Conclusions:**

These results underscored the individual and joint effects of TyG and CRP upon COPD risk, indicating their usefulness as biomarkers for early risk assessment.

**Supplementary Information:**

The online version contains supplementary material available at 10.1186/s12944-025-02732-1.

## Background

As a globally prevalent chronic condition, COPD manifests as persistent airway inflammation and progressive airflow obstruction, contributing significantly to mortality rates and global disease burden [1]. The latest data from the World Health Organization (WHO) revealed that nearly 3.5 million deaths were attributed to COPD in 2021 with a global prevalence of approximately 10.3%, which has undoubtedly imposed a heavy burden on the healthcare systems [2]. The condition’s vague clinical presentation often delays diagnosis until advanced stages. Hence, investigating the underlying causes of COPD is crucial for mitigating its impact on morbidity and mortality [3]. Despite established associations with cigarette smoking, environmental pollutants, and genetics, the specific causes of COPD are still unclear, and its pathogenesis is not fully explained [4]. This uncertainty highlights the need for more work exploring possible contributions.

In recent years, studies have shown that metabolic disturbances may promote the development of COPD, further expanding understanding of its etiological factors [5, 6]. Clinical evidence indicates that abnormalities in glucose metabolism are not only common in COPD patients, but also associated with worsening of lung tissue function [7]. Insulin resistance has increasingly been recognized as a key feature linking metabolic disturbances to chronic diseases [8]. Recent studies have identified the triglyceride-glucose index (TyG) as a recognized indicator of metabolic or cardiovascular diseases via routine clinical evaluation [9]. Emerging evidence suggests that TyG shows diagnostic potential during preclinical respiratory assessments. According to a recent observational study, increased TyG levels are linked to a greater incidence of exertional dyspnea, and a positive relationship with lung dysfunction [10]. Another cross-sectional study briefly analyzed the distribution of TyG across sex and its association with COPD [11]. However, existing studies have focused mainly on the impact of TyG with lung function, while evidence from large-scale cohort studies remains limited. Consequently, the investigation of TyG as a potential risk factor may give new insights into the pathogenesis of COPD.

Furthermore, chronic inflammation induced by insulin resistance is also believed to be a factor in lung tissue damage and remodeling, which leads to an abnormal decline in lung function [12]. Inflammation is thought to underlie lung tissue remodeling and worsening airflow obstruction in individuals with COPD [13]. As a commonly examined biomarker of systemic inflammation, higher CRP levels are related to greater risk of developing COPD, as well as worse clinical outcomes such as exacerbations and mortality [14, 15]. Higher levels of CRP are frequently observed in those diagnosed with COPD, underscoring inflammatory component of the disease [15, 16].

Moreover, the elevation of TyG is often associated with increased inflammatory responses, including higher CRP level. Because of their respective roles as risk factors for chronic diseases, researchers have recently started to investigate how TyG and CRP interact to increase the potential risk [17]. These effect of higher TyG and CRP levels has already been discussed in cardiovascular diseases [18]; however, an in-depth exploration of their effects is still lacking in COPD. Therefore, this study aims to explore the independent and joint effects of TyG and CRP for COPD risk using long-term cohort data from the UK Biobank. By investigating these associations, we hope to identify novel indicators that could facilitate the early detection of COPD, thereby establish a theoretical basis for its prevention efforts.

## Methods

### Study population

Data were derived from the UK Biobank, a longitudinal cohort in the United Kingdom with over half a million participants. At baseline, participants aged 40–69 years from 22 research centers were recruited from 2006 to 2010 [19]. During recruitment, participants underwent an in-person assessment that included a touchscreen questionnaire, physical examinations, and blood sample collection by trained nurses.

In this study, people with airway obstruction were excluded initially [19], encompassing individuals with clinical diagnosed COPD [20], participants who indicated a history of emphysema or chronic bronchitis [20], and those with the ratio of forced vital capacity (FVC) to forced expiratory volume in 1 s (FEV_1_) less than the lower limit of normal (LLN) [21]. Subsequently, we also excluded individuals whose triglyceride (TG), fasting blood glucose (FBG), and CRP levels were not measured (*n* = 67,124). Finally, 385,523 participants were included at baseline (Figure S1).

### Measurement of TyG and CRP

The formula for calculating TyG was as follows [22, 23]:

$$\text{TyG=} \; \text{ln}\;\text{(TG(mg/dL)} \times \text{FBG (mg/dL)}\text{/2)}$$

Baseline TG and FBG were measured by glycerol-3-phosphate oxidase-peroxidase analysis and hexokinase analysis on automatic biochemical analyzer Beckman Coulter AU5800. The units of measurement were mmol/L, which were converted to mg/dL. Besides, CRP was measured by immunoturbidimetric-high sensitivity analysis, with a detection range of 0.08-80.00 mg/L.

### Definition of outcome

New-onset COPD cases were identified by linking data with the National Health Service (NHS) health care registries, utilizing censoring dates of 30 September 2021 for England, 31 July 2021 for Scotland, and 28 February 2018 for Wales. Data regarding the date and causes of death Subsequent to COPD diagnosis were obtained from national death registries, with availability up to 30 September 2021 for England and Wales, and through 31 October in Scotland. Diagnostic information was classified using the 10th edition of the International Classification of Diseases (ICD-10), with COPD defined by codes J40-J44 [20].

### Covariate ascertainment

Covariates were collected from baseline database, including demographic characteristics (age, sex, race, and socioeconomic status), daily behaviors (smoking, drinking, passive smoking, physical activity, and nutritional habits), environmental exposure (including PM_2.5_, high-risk occupation for COPD), personal history of asthma and diabetes, and family history of respiratory diseases. Moreover, lipid biochemical indexes were included in the analysis. Total cholesterol (TC, mmol/L) levels were measured by using the cholesterol oxidase-peroxidase enzymatic colorimetric method. Low-density lipoprotein (LDL) and high-density lipoprotein (HDL) were both quantified through enzymatic methods and are expressed in mmol/L.

In detail, body mass index (BMI, kg/m^2^) was defined as weight (kg) divided by the square of height in meters. Physical activity level was quantified as metabolic equivalent task hours per week (MET-hours/week) for moderate activity [24]. PM_2.5_ exposure was evaluated for each center using land use regression utilizing 2010 data; meanwhile, high-risk occupations for COPD were selected by participants from predefined options in the questionnaire [19]. Baseline asthma was defined according to self-reported information and hospital admissions marked with J45-J46. Moreover, diabetes was defined as follows: (1) diagnosed before recruitment; (2) self-reported use of diabetes medications; (3) registered in the hospital records as diabetic; or (4) hemoglobin A1c (HbA1c) levels ≥ 6.5% [25]. The diagnosed diabetes was coded as E10-E14.

### Statistical analysis

Person-years were accrued starting from the assessment date, with follow-up censored at whichever came first: being diagnosed with COPD, passing away, becoming lost to follow-up, or reaching the deadline of this study. Continuous variables are reported as the means ± standard deviations (SDs), and categorical variables are presented as frequencies and percentages (n, %). Statistical differences between participants with and without TyG and CRP data were evaluated by calculating the standardized mean difference (SMD), where values over 0.2 were interpreted as meaningful differences.

Besides, hazard ratios (HRs) and 95% confidence intervals (CIs) for COPD risk were derived from Cox proportional hazard models. Three models were developed to estimate the associations of TyG and CRP with COPD risk: (1) Model 1 was adjusted for age, sex, and race; (2) Model 2 plus adjusted for socioeconomic status, daily lifestyles, environmental exposure, baseline asthma, and family history of respiratory diseases; and (3) Model 3 further included BMI, baseline diabetes, and biochemistry indexes (TC, HDL, and LDL). We divided TyG and CRP as quintiles (Q1 to Q5), and defined the lowest quintile as the reference to show the associations. Besides, restricted cubic spline (RCS) curves and 3D-surface plots were generated to visualize the dose-response relationships for TyG and CRP on COPD risk. The List of covariates incorporated in the RCS was consistent with that in Model 3.

To analyze the joint effect of TyG and CRP, we first performed ROC curves to determine the optimal cut-off values for both variables based on their area under the curves (AUC). TyG and CRP were categorized into high and low groups (< cut-off, ≥ cut-off), and a four-level categorical variable was constructed according to their optimal cut-off values identified through ROC curves: (1) low TyG and low CRP (defined as the reference); (2) low TyG and high CRP; (3) high TyG and low CRP; and (4) high TyG and high CRP. Meanwhile, Kaplan-Meier (KM) curves were plotted to illustrate the cumulative hazard across TyG-CRP joint categories, and the log-rank test was used to examine group-level differences on COPD risk. To estimate the magnitude of the joint effect, we Subsequently constructed Cox models adjusted for the complete set of covariates specified in Model 3.

Stratified analysis was conducted to evaluate the association between the joint TyG-CRP and COPD risk across different subgroups. Specifically, multiplicative interaction terms between the joint variable and subgroup variables (e.g., age, sex, smoking, passive smoking, BMI, physical activity, baseline asthma, baseline diabetes, and family history of respiratory diseases) were included in Cox models. The likelihood ratio tests were conducted to compare models with and without interaction terms to assess the significance to effect modification. Moreover, the Baron & Kenny method was applied to fit the mediation and outcome models, to evaluate both the direct effect of TyG on COPD risk and its indirect effect mediated by CRP. Meanwhile, the potential confounders in Cox models were also adjusted in the mediation analysis.

Finally, sensitivity analysis was performed to validate the robustness of results, which was performed as follows: (1) excluding individuals who developed COPD during the first 2 years of follow-up; (2) excluding people self-reporting poor health status; and (3) excluding those with extremely high CRP levels (> 10 mg/L). All analyses were performed by using R software (version 4.4) and SAS software (version 9.4). Statistical significance was determined using two-sided tests, with a threshold of *P* < 0.05.

## Results

### Baseline characteristics

No notable differences were found between included and excluded participants, as all SMDs < 0.2 (Table S1). Over a median follow-up period of 12.28 years, a total of 10,515 COPD cases were diagnosed. The average age was 56.5 years at baseline, with females comprising almost 54.2%. We listed baseline characteristics categorized by TyG, and found the proportion of females were decreased from Q1 (< 6.63) to Q5 (≥ 7.59), whereas BMI increased with higher TyG level. Participants with increased TyG had higher CRP levels, proportion of asthma, and tended to frequent smoking. The proportion of tertiary educated and frequency of physical activity was lower among participants, and a total of 23,050 people had baseline diabetes (6.0%).

After classifying participants into five groups based on quintiles of CRP, we observed that the proportions of baseline diabetes, asthma, and family history of respiratory diseases increased among individuals with higher CRP level (Table S2). Furthermore, we compared the baseline characteristics between participants excluded solely due to FEV_1_/FVC < LLN and those excluded based on clinical diagnosis or self-reported respiratory disease among individuals with baseline airway obstruction. Apart from differences in alcohol consumption frequency and a family history of respiratory diseases, no significant disparities were observed (Table S3).

### Associations of tyg, CRP with COPD risk

Among the total participants, we observed that comparing with the lowest quintile (Q1), higher TyG levels were associated with elevated COPD risk in each model (*P*_for trend_ < 0.001). Upon adjustment for covariates, the risk also increased by approximately 25% when TyG ≥ 7.59 (HR = 1.25, 95% CI: 1.15–1.36). Besides, higher risk was also observed with higher CRP levels in Model 3 (Q2 vs. Q1: HR = 1.22, 95% CI: 1.12–1.33; Q3 vs. Q1: HR = 1.48, 95% CI: 1.37–1.61; Q4 vs. Q1: HR = 1.79, 95% CI: 1.66–1.94; Q5 vs. Q1: HR = 2.28, 95% CI: 2.11–2.46, respectively) (Table 1).

**Table 1:** Demographic characteristics of participants at baseline (*N*=385,523)

	TyG				
	Q1 (< 6.63)	Q2 (6.63-)	Q3 (6.95-)	Q4 (7.24-)	Q5 (≥ 7.59)
Participants, n	77,105	77,104	77,105	77,105	77,104
Females, n (%)	52,966 (68.69)	47,173 (61.18)	42,255 (54.80)	37,198 (48.24)	29,538 (38.31)
White ethnicity, n (%)	71,604 (92.87)	72,888 (94.53)	73,213 (94.95)	73,396 (95.19)	73,018 (94.70)
Age, years, mean ± SD	53.83 ± 8.27	56.32 ± 8.07	57.23 ± 7.90	57.62 ± 7.77	57.42 ± 7.75
Townsend deprivation index, mean ± SD	-1.41 ± 3.05	-1.48 ± 2.99	-1.45 ± 2.99	-1.41 ± 3.03	-1.20 ± 3.12
College or university degree, n (%)	30,594 (39.68)	26,678 (34.60)	24,730 (32.07)	23,238 (30.14)	21,490 (27.87)
BMI, kg/m^2^, mean ± SD	25.05 ± 3.94	26.49 ± 4.40	27.61 ± 4.57	28.55 ± 4.62	29.75 ± 4.73
Physical activity, MET-hours/week, mean ± SD	14.70 ± 18.73	14.54 ± 18.82	14.30 ± 18.76	13.90 ± 18.50	13.13 ± 18.09
Current smoking, n (%)	5672 (7.36)	6300 (8.17)	6832 (8.86)	7668 (9.94)	9220 (11.96)
Passive smoking, n (%)	13,791 (17.89)	13,940 (18.08)	14,366 (18.63)	14,869 (19.28)	16,159 (20.96)
Alcohol consumption almost daily, n (%)	35,470 (46.00)	34,497 (44.74)	33,467 (43.40)	32,902 (42.67)	31,616 (41.00)
Vegetable intake, n (%)	70,408 (91.31)	69,584 (90.25)	69,033 (89.53)	68,331 (88.62)	66,821 (86.66)
Fresh fruit intake, n (%)	729,93 (94.67)	72,775 (94.39)	725,11 (94.04)	71,976 (93.35)	70,881 (91.93)
Oily fish intake, n (%)	69,707 (90.41)	69,141 (89.67)	68,430 (88.75)	67,908 (88.07)	66,646 (86.44)
Asthma, n (%)	7611 (9.87)	7703 (9.99)	7729 (10.02)	7907 (10.25)	8013 (10.39)
Diabetes, n (%)	1587 (2.06)	2044 (2.65)	2923 (3.79)	4560 (5.91)	11,936 (15.48)
Family history of respiratory diseases, n (%)	9904 (12.84)	11,204 (14.53)	11,665 (15.13)	12,060 (15.64)	12,290 (15.94)
High-risk occupations of COPD, n (%)	1485 (1.93)	1378 (1.79)	1417 (1.84)	1501 (1.95)	1448 (1.88)
PM_2.5_, μg/m^3^, mean ± SD	9.97 ± 1.04	9.93 ± 1.01	9.93 ± 1.00	9.94 ± 1.00	9.97 ± 1.01
HDL, mmol/L, mean ± SD	1.69 ± 0.40	1.56 ± 0.37	1.45 ± 0.34	1.34 ± 0.30	1.19 ± 0.27
LDL, mmol/L, mean ± SD	3.19 ± 0.72	3.47 ± 0.78	3.63 ± 0.84	3.76 ± 0.88	3.78 ± 0.95
TC, mmol/L, mean ± SD	5.32 ± 0.99	5.60 ± 1.05	5.74 ± 1.10	5.87 ± 1.15	5.99 ± 1.28
CRP, mg/L, mean ± SD	1.86 ± 4.10	3.30 ± 4.28	2.63 ± 4.39	2.86 ± 4.31	3.12 ± 4.14

*Abbreviations*: *BMI* body mass index, *MET* metabolic equivalent task, *HDL* high-density lipoprotein, *LDL* low-density lipoprotein, *TC* total cholesterol, *CRP* C-creative protein, *TyG* triglyceride-glucose index

Moreover, the nonlinear trends of TyG and CRP with COPD risk were illustrated in RCS plots (Figure S2). Both TyG and CRP exhibited a nonlinear positive association with COPD risk, with all *P*_for nonlinearity_ < 0.001. When considering TyG and CRP simultaneously, the 3D-surface also showed an increasing trend in both higher levels (Figure S3).

#### Joint effect analysis

**Fig. 1:**
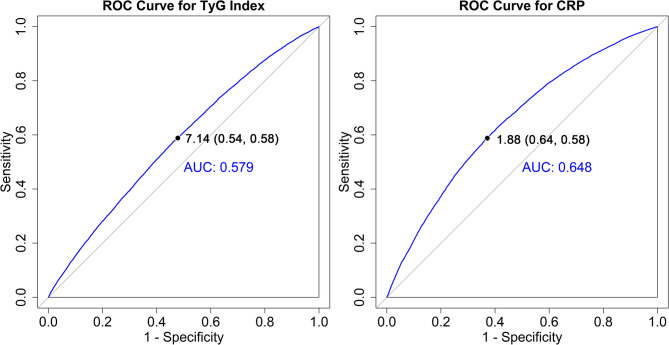
The optimal cut-off values of TyG and CRP for evaluating the risk of COPD among participants

**Fig. 2:**
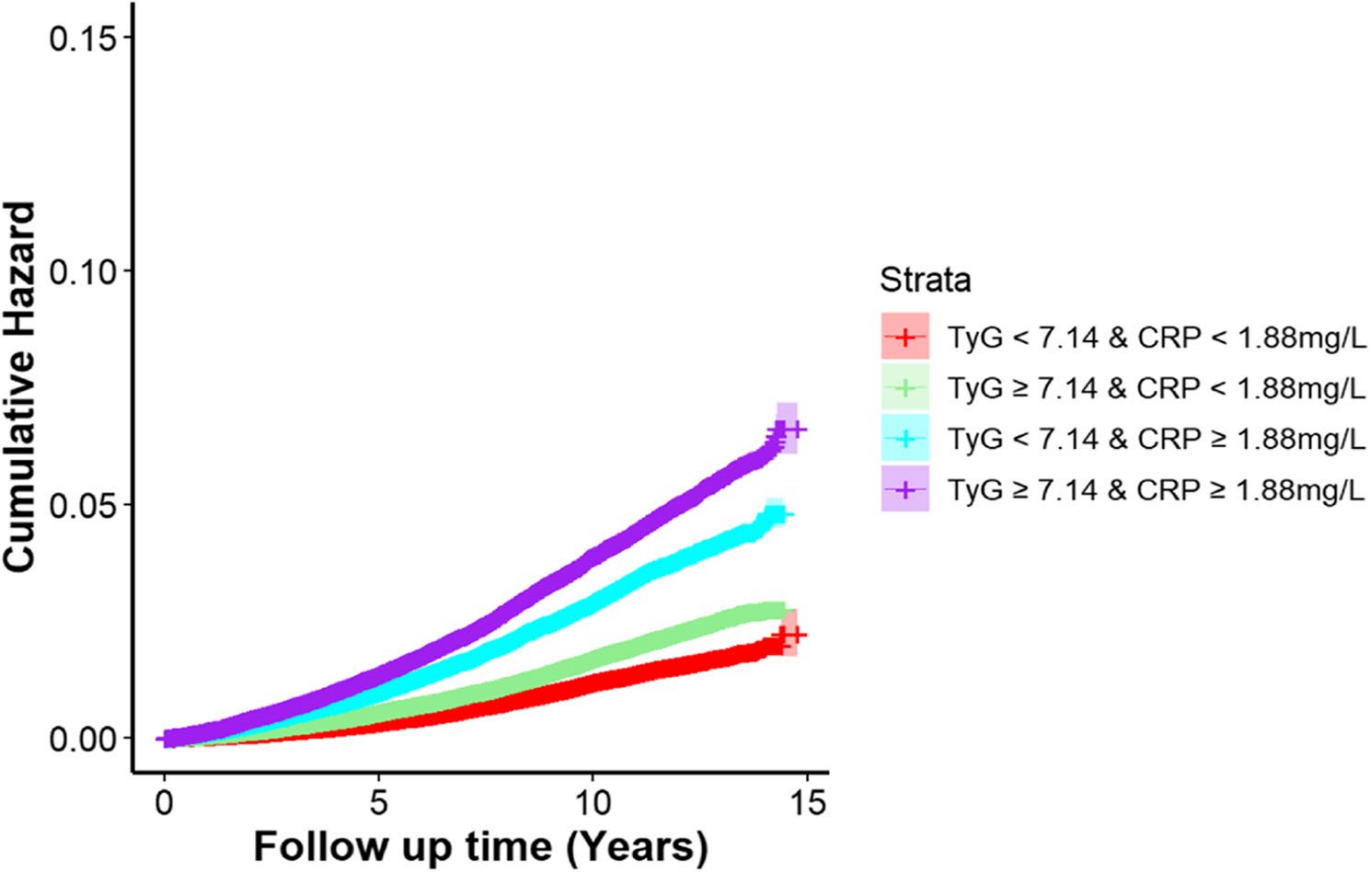
Kaplan-Meier plot for the cumulative hazard of COPD by TyG and CRP levels

To evaluate the joint effect, we firstly categorized TyG and CRP based on their optimal cut-off values. As shown in Fig. 1, the cut-off values were 7.14 and 1.88 mg/L for TyG and CRP, with the AUC values of 0.579 and 0.648, respectively. We constructed a four-category joint variable derived from the cut-off of TyG (< 7.14, ≥ 7.14) and CRP (< 1.88 mg/L, ≥ 1.88 mg/L), to assess their combined effect on COPD risk. When we combined TyG and CRP, we found baseline characteristics of subgroups were similar, as most of SMDs < 0.2 (Table S4) (Fig. 2).

**Fig. 3:**
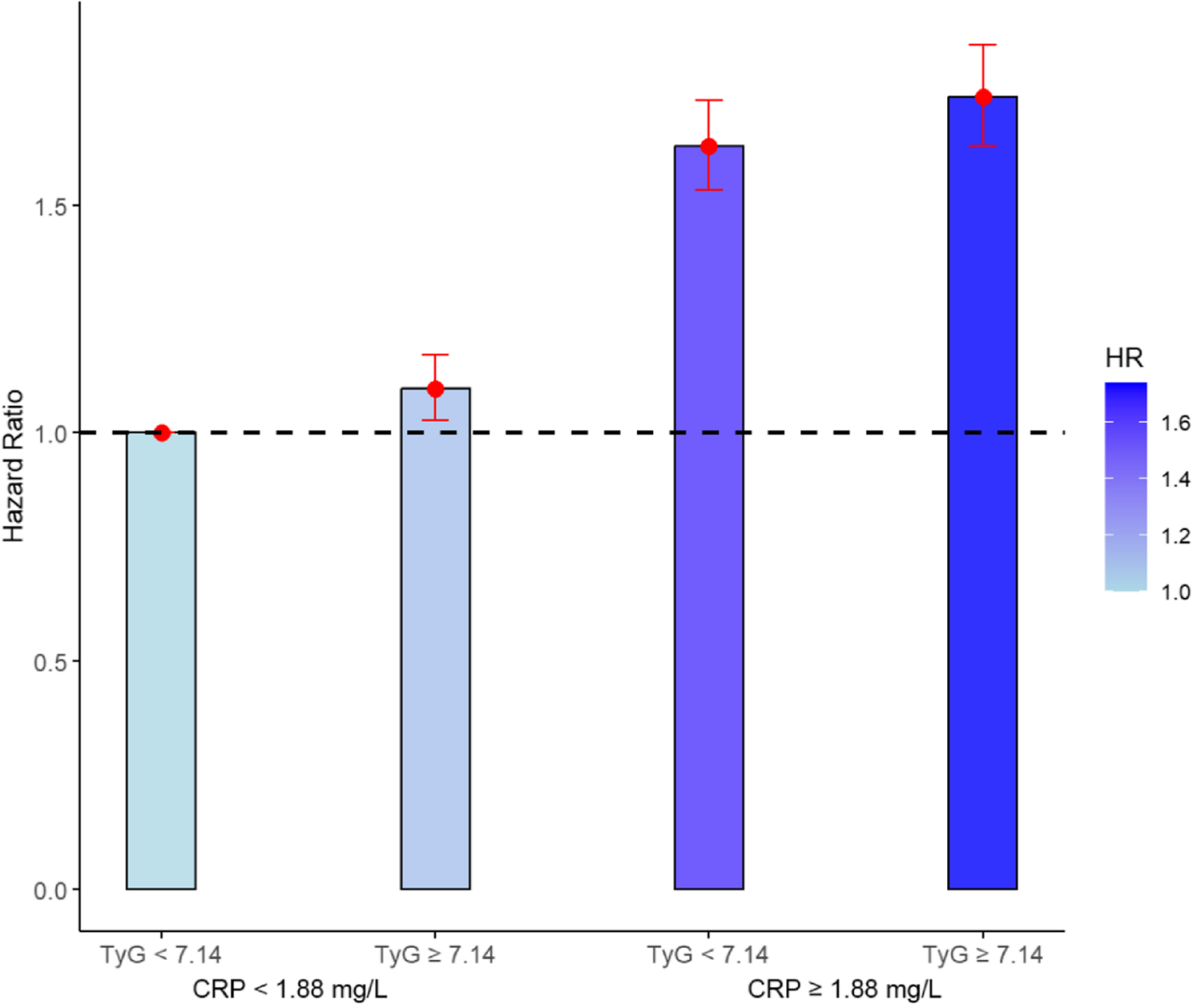
The joint effect for TyG and CRP on the risk of COPD

Over the follow-up period, the cumulative hazard of COPD differed significantly across the TyG-CRP joint exposure groups (*P*_for log−rank_ < 0.05). Compared with reference group, the subgroup with higher TyG (≥ 7.14) and low CRP (< 1.88 mg/L) showed a modest increase in cumulative hazard (Table 2). In contrast, individuals with higher CRP and TyG < 7.14 exhibited a more pronounced increase, suggesting a stronger effect of CRP levels. Besides, the highest cumulative hazard was observed in higher levels of both TyG and CRP. Furthermore, a significant increasing trend in COPD risk was observed as both TyG and CRP levels increased by adjusting all covariates in Cox regression model. In details, the highest risk (HR = 1.74, 95% CI: 1.63–1.85) was observed among the group of CRP ≥ 1.88 mg/L and TyG ≥ 7.14 (Fig. 3).

**Table 2:** The associations of TyG and CRP on the risk of COPD in the UK Biobank

	No. of COPD cases	Model 1		Model 2		Model 3	
		HR (95%CI)	*P*	HR (95%CI)	*P*	HR (95%CI)	*P*
TyG							
Q1 (< 6.63)	1,313	ref		ref		ref	
Q2 (6.63-)	1,806	1.13 (1.05-1.22)	< 0.001	1.07 (0.99-1.14)	< 0.001	1.12 (1.04-1.20)	< 0.001
Q3 (6.95-)	2,070	1.20 (1.12-1.29)	< 0.001	1.06 (0.99-1.13)	< 0.001	1.13 (1.05-1.23)	< 0.001
Q4 (7.24-)	2,389	1.34 (1.25-1.43)	< 0.001	1.10 (1.03-1.17)	< 0.001	1.19 (1.10-1.28)	< 0.001
Q5 (≥ 7.59)	2,937	1.65 (1.54-1.76)	< 0.001	1.20 (1.12-1.28)	< 0.001	1.25 (1.15-1.36)	< 0.001
*P*_for trend_		< 0.001		< 0.001		< 0.001	
CRP (mg/L)							
Q1 (< 0.55)	906	ref		ref		ref	
Q2 (0.55-)	1,292	1.29 (1.18-1.40)	< 0.001	1.16 (1.06-1.26)	0.001	1.22 (1.12-1.33)	< 0.001
Q3 (1.01-)	1,877	1.73 (1.60-1.88)	< 0.001	1.39 (1.28-1.50)	< 0.001	1.48 (1.37-1.61)	< 0.001
Q4 (1.73-)	2,587	2.35 (2.18-2.54)	< 0.001	1.64 (1.52-1.77)	< 0.001	1.79 (1.66-1.94)	< 0.001
Q5 (≥ 3.28)	3,853	3.42 (3.16-3.70)	< 0.001	2.12 (1.97-2.28)	< 0.001	2.28 (2.11-2.46)	< 0.001
*P*_for trend_		< 0.001		< 0.001		< 0.001	

### Stratified and sensitivity analysis

Within stratified analysis, the association between the joint TyG-CRP variable and COPD risk varied across different Subgroups. For participants aged younger than 60 years, the joint HR for COPD risk was higher than those who aged ≥ 60 years (*P*_for interaction_ < 0.001). A more pronounced effect modification of the association was also observed among current smokers and passive smokers, and the risk was also more significant in individuals with BMI < 25.0 kg/m^2^ (all *P*_for interaction_ < 0.05). In addition, among participants with low levels of physical activity (< 8 MET-hours/week), the joint risk associated with elevated CRP (≥ 1.88 mg/L) and TyG (≥ 7.14) was higher compared to those with higher activity levels (*P*_for interaction_ =0.002). Notably, the joint effect was more significant in those without baseline diabetes, asthma, or family history of respiratory diseases (Figure S4).

As shown in Table S5, TyG remained significantly positive association with COPD risk after excluding various subgroups in sensitivity analysis (all *P*_for trend_ < 0.05). Nonlinear dose-response curves also illustrated the positive associations between TyG and COPD risk (Figure S5). Moreover, we observed that regardless of TyG level, higher CRP could significantly increase COPD risk (Table S6).

### Mediation analysis

**Fig. 4:**
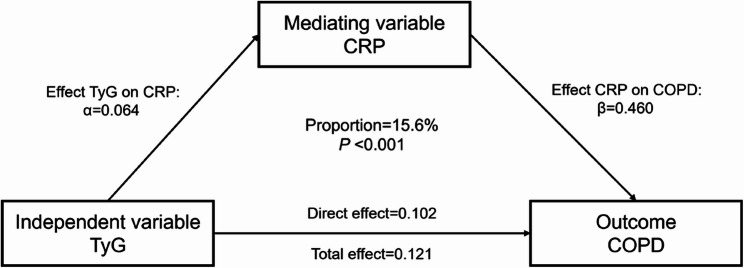
The mediation effect of CRP in the relationship between TyG and COPD risk

Figure 4 Summarized the mediation effects Linking TyG and CRP to COPD risk. Higher level of CRP mediated approximately 15.6% of association between TyG and COPD risk (*P* < 0.001), with the direct effect coefficient was 0.012. In the sensitivity analysis (Figure S6), the mediation effect was slightly lower in individuals without abnormal CRP levels (14.8%) and higher in baseline healthy participants (23.0%).

## Discussion

While the role of TyG as a metabolic marker is well established, its linkage to inflammation has garnered increasing attention. As a representative inflammatory biomarker, CRP provides further insights into this relationship [26]. However, their combined effect on COPD risk has not yet been investigated. Our findings extend existing evidence by demonstrating that the combined elevation of TyG and CRP significantly increases COPD risk. Notably, we demonstrated the joint effect of both indexes, with the highest risk observed when both markers were elevated. Furthermore, our results indicated a mediation relationship between TyG and CRP with COPD risk.

The association between metabolic indicators and chronic lung diseases is increasingly being explored. The main advantage of TyG as a metabolic marker is the capacity to jointly indicate insulin resistance and lipid metabolism, which helps to capture the characteristics of metabolic dysfunction. Liu et al. reported higher TyG levels were positively associated with increased risk of acute myocardial infarction and sudden cardiac arrest among Chinese [27]. In 2022, a Swedish follow-up study identified elevated TyG as a novel biomarker for predicting COPD risk, reporting a HR of 1.72 (95% CI: 1.41–2.09) [28]. Our results also confirmed that as TyG levels increased, the risk of COPD consistently raised, providing new evidence for the relationship between TyG as a metabolic indicator and COPD risk.

The exacerbation of airway inflammation may initially stem from disruptions in insulin signaling pathways. Insulin resistance enhances oxidative stress and activates inflammatory pathways (NF-κB and MAPK pathway) in turn, exacerbating airway inflammation [29]. Meanwhile, insulin resistance impairs normal immune cell function in the lungs and dysregulated immunity, thus aggravating airway inflammation and tissue damage [30]. Recently, Ruan et al. highlighted the insulin metabolic network is involved in airway inflammation, and poor prognosis of COPD [31]. Ferreira et al. demonstrated in diabetic mice that insulin could influence airway alterations through its effects on immune regulation [32]. These mechanisms proposed how insulin resistance implicates in COPD pathogenesis, thus stressing the relevance of correction of metabolic disturbances as a prospective strategy for COPD prevention and management.

On the other hand, CRP further facilitates to pulmonary dysfunction by mediating the migration and functional stimulation of innate immune cells (such as neutrophils or macrophages) [33]. These activated phagocytes secrete matrix-degrading proteases and inflammatory mediators that collectively induce alveolar epithelial injury, compromise respiratory membrane integrity, and ultimately lead to bronchoconstriction [34]. Furthermore, inflammation can induce oxidative stress, which exacerbates lung function decline and promotes the transcription of additional inflammatory mediators, thereby perpetuating the vicious cycle of inflammation and oxidative damage [35]. The combined assessment of the both indexes has already been applied in the risk prediction and prognosis of cardiovascular diseases and cancers, but its impact on COPD risk still requires further evidence. We identified a significant association on the joint effect, with a similar trend observed on other diseases from previous study [36]. This also highlights the universality of the combined index for estimating risk of diseases, which can aid in the early warning and identification of COPD, providing important evidence for stratified screening and management of high-risk populations.

In this study, we utilized cut-off values to categorize TyG and CRP levels, which allowed for a more clinically relevant stratification of risk. Currently, most studies focused on adverse cardiovascular events like atherosclerosis and aortic stenosis [17, 37], with lack of evidence on COPD risk. Additionally, the optimal cut-off values of TyG vary significantly across different diseases, and some researchers use the median or mean of TyG as the basis for grouping, resulting in a lack of a unified and clear standard [36]. However, despite the lower AUC value in this study, TyG still showed potential in identifying individuals at risk of COPD. On the other hand, while the critical value of CRP is well-documented in acute respiratory diseases, its optimal cut-off for COPD onset remains controversial. According to previous meta-analysis in 2017, CRP had often been associated with acute exacerbations or mortality among COPD patients, leading to higher cut-off value Such as 3 mg/L, to characterize poor prognosis [15]. The cut-off values determined using the ROC curve can more effectively differentiate individuals with different risk levels, providing more targeted reference for the early detection of COPD and clinical decision-making. Future studies are need to combine clinical features and large-scale population data to validate its clinical applicability.

In terms of stratified analysis, we observed that the combined risk of TyG and CRP for COPD was more significant among smokers or passive smokers. It is well-known that smokers increase oxidative stress and inflammation and damage airways which can cause impaired lung function. Metabolic disorders are one of the major consequences of smoking [38]. Interestingly, the joint effect appeared stronger among individuals without asthma or a family history of respiratory disease. This may be explained by the reduced interference from genetic or existing conditions in those without such history, which allows the metabolic and inflammatory effects to emerge more prominently. Meanwhile, we also observed an opposite trend when CRP < 1.88 mg/L and TyG ≥ 7.14 among baseline diabetes patients. Diabetes cases often already have insulin resistance [39], which may eliminate the effect of TyG. On the other hand, participants with baseline diabetes may be receiving medication (such as insulin injection or hypoglycemic drugs), which can alter the impact of TyG and CRP levels on COPD risk. When CRP is lower, indicating the absence of significant inflammation, a protective effect may emerge, further reducing COPD risk. In addition, the combined effect was more significant among males, and people younger than 60 years old, and those with lower BMI. Due to higher smoking rate in males, the impact of TyG and CRP on COPD risk may be more pronounced, leading to stronger effect compared to females [40]. Among those who are younger and have lower BMI, the physiological response to metabolic and inflammatory disturbances may be more pronounced, thereby amplifying their risk of COPD [41].

These results point to a possible mediating pathway involving CRP in the Link between TyG and COPD risk. Li et al. explored the mediating roles of TyG and CRP in the association between obesity and colorectal cancer risk in 93,659 participants, confirming that both CRP and TyG independently or synergistically increase the risk [42]. Chen et al. have found that CRP has also been proposed to mediate the association between TyG levels and lung function [43]. However, we did not proceed with an additional mediation analysis involving TyG as a potential intermediate factor linking CRP to COPD risk, given that TyG reflects long-term metabolic status, whereas inflammation is a more immediate clinical response. Therefore, we believe that it is more plausible for changes in metabolic characteristics to mediate COPD onset through inflammation. In the sensitivity analysis, the results remained stable, which indirectly supports the rationale of using CRP as a mediator in the pathway through which TyG contributes to COPD.

### Strengths and limitations

There were several strengths of this study. First, it utilized data from the UK Biobank, providing a robust basis for the findings. The follow-up was long term, which allowed for correct assessment of the associations of baseline TyG and CRP on COPD risk, which brought important insights into possible causal relationships. Moreover, this study extended previous evidence by demonstrating a joint impact of TyG and CRP on COPD, while mediation analysis suggested that CRP partially mediates for the association.

Notwithstanding the above advantages, some limitations could not be avoided. The potential biases and confounding factors cannot be eliminated completely, for the causal conclusions still remained uncertain. Second, we used baseline measurement data only, and did not consider the possible dynamic effects of TyG and CRP over time, which could possibly impede a better understanding of their long-term effects. Moreover, the potential impact of pharmacological interventions, including therapies for diabetes or inflammation, was not taken into account in this analysis. Additionally, the incidence of COPD was identified solely using ICD-10 codes, which carries a risk of misclassification. Future research should include multi-time point studies and trajectory analyses to clarify how TyG and CRP change over time during follow-up. Moreover, the cut-off values for TyG and CRP were determined by ROC curve analysis within the same cohort used for outcome modeling. This approach may lead to overfitting and optimistic estimates of risk prediction. Due to resource limitations, internal validation methods such as bootstrapping or cross-validation were not performed. Although statistically significant, the AUC values suggest limited discriminatory power of TyG and CRP when used individually. Despite adjustment for covariates, potential confounding cannot be entirely ruled out, and the mediation analysis results may still be biased. While the relationship between TyG and CRP with COPD risk was demonstrated, it does not delve deeply into the specific molecular mechanisms, leaving a gap in understanding the precise pathways through which these factors influence the disease. Furthermore, it needs in-depth research for underlying mechanisms to confirm our results.

## Conclusions

Overall, this study indicated that both TyG and CRP, whether individually or in combination, were associated with higher COPD risk. Additionally, CRP mediated the association between TyG and COPD risk, indicating a potential link involving metabolic dysfunction and inflammation status. These findings highlight the value of incorporating TyG and CRP as clinical biomarkers for risk stratification of COPD.

## Supplementary Information

Supplementary Material 1.

## Data Availability

UK Biobank data is available at https://www.ukbiobank.ac.uk/. This research was conducted under application number 84525.

## Abbreviations

TyG: Triglyceride-glucose index
ICD-10: the 10th edition of the International Classification of Diseases
CRP: C-reactive protein
MET: Metabolic equivalent
FEV_1_: Forced expiratory volume in the first second
LLN: Lower limit of normal
RCS: Restricted cubic spline
FVC: Forced vital capacity
COPD: Chronic obstructive pulmonary disease
Mets: Metabolic syndrome
BMI: Body mass index.
