# Supplementary material for: The joint effect of triglyceride-glucose index and C-reactive protein levels on the risk of chronic obstructive pulmonary disease: a prospective cohort study
Source: Lipids Health Dis. 2025 Oct 6;24:309. doi: 10.1186/s12944-025-02732-1 (PMC12502271; doi:10.1186/s12944-025-02732-1)
Supplement: Supplementary file 1 — Supplementary Material 1. [file 12944_2025_2732_MOESM1_ESM.pdf]

Table S1. Baseline characteristics of participants who included or excluded in this study.

Baseline characteristics	Included (n=385,523)	Excluded (n=67,124)	SMD*
Age, years, mean ± SD	56.49 ± 8.08	56.33 ± 8.11	0.020
Females, n (%)	209,130 (54.2)	38,926 (58.0)	0.076
White ethnicity, n (%)	364,119 (94.4)	62,584 (93.2)	0.075
Townsend deprivation index, mean ± SD	-1.39 ± 3.04	-1.28 ± 3.10	0.036
College or university degree, n (%)	126,730 (32.9)	20,922 (31.2)	0.193
BMI, kg/m^2, mean ± SD	27.49 ± 4.75	27.46 ± 4.84	0.007
Physical activity, moderate MET-hours/week, mean ± SD	14.12 ± 18.59	13.62 ± 17.97	0.027
Current smoking, n (%)	27,620 (7.2)	4,898 (7.3)	0.066
Passive smoking, n (%)	73,125 (19.0)	13,559 (20.2)	0.038
Alcohol intake daily or almost daily, n (%)	143,290 (37.2)	28,364 (42.3)	0.085
Asthma, n (%)	38,963 (10.1)	6,885 (10.3)	0.005
Family history of respiratory diseases, n (%)	57,123 (14.8)	9,988 (14.9)	0.043
High-risk occupations of COPD, n (%)	7,229 (1.9)	1,223 (1.8)	0.027
$PM_{2.5}$, $\mu g/m^3$, mean ± SD	9.95 ± 1.01	9.98 ± 1.01	0.036
HDL, mmol/L, mean ± SD	1.45 ± 0.38	1.42 ± 0.17	0.098
LDL, mmol/L, mean ± SD	3.57 ± 0.87	3.53 ± 0.65	0.049
TC, mmol/L, mean ± SD	5.70 ± 1.04	5.68 ± 0.87	0.018

Abbreviations: BMI: body mass index; MET: metabolic equivalent task; HDL: high-density lipoprotein; LDL: low-density lipoprotein; TC: total cholesterol; SMD, standardized mean difference.

* SMD indicated a potentially relevant difference between the groups.

Table S2. Baseline characteristics of participants according to quintile of CRP (N=385,523).

	CRP (mg/L)				
	Q1 (< 0.55)	Q2 (0.55-)	Q3 (1.01-)	Q4 (1.73-)	Q5 (≥ 3.28)
Participants, n	77,601	76,105	77,378	77,459	77,070
Females, n (%)	43,033 (55.6)	38,730 (51.0)	39,445 (51.0)	41,530 (53.6)	46,392 (60.2)
White ethnicity, n (%)	73,307 (94.5)	72,006 (94.7)	73,238 (94.7)	73,232 (94.5)	72,336 (93.9)
Age, years, mean ± SD	54.66 ± 8.15	56.18 ± 8.07	56.96 ± 8.02	57.37 ± 7.90	57.26 ± 7.93
Townsend deprivation index, mean ± SD	-1.59 ± 2.93	-1.58 ± 2.94	-1.50 ± 2.99	-1.32 ± 3.06	-0.96 ± 3.23
College or university degree, n (%)	32,958 (42.5)	27,311 (35.9)	25,018 (32.3)	21,989 (28.4)	19,454 (25.2)
BMI, kg/m^2, mean ± SD	24.54 ± 3.31	26.23 ± 3.59	27.43 ± 3.94	28.70 ± 4.41	30.56 ± 5.75
Physical activity, MET-hours/week, mean ± SD	14.92 ± 18.94	14.80 ± 19.02	14.43 ± 18.83	13.79 ± 18.49	12.65 ± 17.53
Current smoking, n (%)	5,414 (7.0)	5,768 (7.6)	6,678 (8.6)	7,938 (10.3)	9,894 (12.8)
Passive smoking, n (%)	13,299 (17.1)	13,833 (18.2)	14,714 (19.0)	15,168 (19.6)	16,111 (20.9)
Alcohol consumption almost daily, n (%)	37,966 (48.9)	35,815 (47.1)	34,502 (44.6)	31,779 (41.0)	27,890 (36.2)
Vegetable intake, n (%)	70,852 (91.3)	68,565 (90.1)	69,308 (89.6)	68,414 (88.3)	67,038 (87.0)
Fresh fruit intake, n (%)	74,140 (95.5)	71,860 (94.4)	72,576 (93.8)	71,899 (92.8)	70,661 (91.7)
Oily fish intake, n (%)	70,056 (90.3)	68,280 (89.7)	68,850 (89.0)	68,090 (87.9)	66,556 (86.4)
Asthma, n (%)	6,422 (8.3)	6,806 (9.0)	7,411 (10.0)	8,302 (10.7)	10,022 (13.0)
Diabetes, n (%)	2,835 (3.7)	3,453 (4.5)	4,242 (5.5)	5,225 (6.8)	7,295 (9.5)
Family history of respiratory diseases, n (%)	9,461 (12.2)	10,412 (13.7)	11,417 (14.8)	12,530 (16.2)	13,303 (17.3)
High-risk occupations of COPD, n (%)	1,342 (1.7)	1,291 (1.7)	1,464 (1.9)	1,470 (1.9)	1,662 (2.2)
$PM_{2.5}$, μg/m^3, mean ± SD	9.93 ± 1.03	9.92 ± 1.01	9.93 ± 1.00	9.96 ± 1.01	10.00 ± 1.02
HDL, mmol/L, mean ± SD	1.57 ± 0.40	1.49 ± 0.39	1.44 ± 0.37	1.39 ± 0.36	1.35 ± 0.35
LDL, mmol/L, mean ± SD	3.41 ± 0.81	3.54 ± 0.85	3.61 ± 0.87	3.66 ± 0.89	3.62 ± 0.90
TC, mmol/L, mean ± SD	5.56 ± 1.08	5.69 ± 1.11	5.75 ± 1.15	5.79 ± 1.17	5.71 ± 1.17
TyG, mean ± SD	6.87 ± 0.52	7.05 ± 0.54	7.15 ± 0.55	7.24 ± 0.56	7.29 ± 0.57

Abbreviations: BMI: body mass index; MET: metabolic equivalent task; HDL: high-density lipoprotein; LDL: low-density lipoprotein; TC: total cholesterol; CRP: C-creative protein; TyG: triglyceride-glucose index.

Table S3. Baseline characteristics between excluded participants with airway obstruction based on LLN criteria (N=49,724).

Baseline characteristics	Without LLN-defined obstruction (n=10,553)	LLN-defined airway obstruction (n=39,095)	SMD*
Age, years, mean ± SD	56.25 ± 9.01	56.32 ± 8.28	0.142
Females, n (%)	5,424 (51.4)	20,075 (51.3)	0.009
White ethnicity, n (%)	10,052 (95.4)	35,818 (91.6)	0.156
College or university degree, n (%)	3,081 (29.2)	11,711 (30.1)	0.164
BMI, kg/m^2, mean ± SD	28.02 ± 5.79	27.51 ± 4.62	0.157
Current smoking, n (%)	944 (8.9)	3,655 (9.3)	0.134
Passive smoking, n (%)	2,357 (22.4)	9,400 (24.0)	0.192
Alcohol intake daily or almost daily, n (%)	3,814 (36.1)	16,729 (42.8)	0.226
Family history of respiratory diseases, n (%)	2,810 (26.6)	7,055 (18.4)	0.244
High-risk occupations of COPD, n (%)	282 (2.7)	1,095 (2.8)	0.013
$PM_{2.5}$, $\mu g/m^3$, mean ± SD	9.98 ± 1.05	10.01 ± 1.09	0.084
HDL, mmol/L, mean ± SD	1.40 ± 0.40	1.45 ± 0.39	0.162
LDL, mmol/L, mean ± SD	3.40 ± 0.93	3.51 ± 0.86	0.124
TC, mmol/L, mean ± SD	5.48 ± 1.22	5.64 ± 1.13	0.135

Abbreviations: BMI: body mass index; MET: metabolic equivalent task; HDL: high-density lipoprotein; LDL: low-density lipoprotein; TC: total cholesterol; SMD, standardized mean difference.

* SMD > 0.2 indicated a potentially relevant difference between the groups.

Table S4. Baseline characteristics among participants according to the joint variable of TyG and CRP*.

Baseline characteristics	CRP < 1.88mg/L		CRP ≥1.88mg/L		SMD
	TyG < 7.14	TyG ≥ 7.14	TyG < 7.14	TyG ≥ 7.14	
No. of participants	145,001	97,526	60,669	82,327	/
Age	55.16 ± 8.22	57.22 ± 7.85	56.60 ± 8.18	57.85 ± 7.66	0.131
Females	90,043 (62.1)	37,130 (38.1)	38,888 (64.1)	43,069 (52.3)	0.301
White ethnicity	136,899 (94.4)	92,533 (94.9)	56,465 (93.1)	78,222 (95.0)	0.172
Townsend deprivation index	-1.55 ± 2.95	-1.54 ± 2.97	-1.18 ± 3.14	-1.08 ± 3.17	0.078
College or university degree	56,316 (38.8)	32,504 (33.3)	17,720 (29.2)	20,190 (24.5)	0.171
BMI	25.20 ± 3.56	27.59 ± 3.82	28.55 ± 5.17	30.63 ± 5.16	0.552
Physical activity	14.91 ± 18.92	14.32 ± 18.86	13.76 ± 18.49	12.72 ± 17.64	0.065
Current smoking	10,377 (7.2)	8,565 (8.8)	6,138 (10.1)	10,612 (12.9)	0.124
Passive smoking	35,256 (24.3)	25,715 (26.4)	16,900 (27.9)	24,173 (29.4)	0.116
Alcohol intake daily or almost daily	52,986 (36.5)	35,647 (36.6)	23,144 (38.2)	31,513 (38.3)	0.145
Family history of respiratory diseases	19,094 (13.2)	13,979 (14.3)	9,726 (16.0)	14,324 (17.4)	0.076
High-risk occupations of COPD	2,536 (1.8)	1,759 (1.8)	1,295 (2.1)	1,639 (2.0)	0.129
Baseline asthma	13,051 (9.0)	8,718 (8.9)	7,442 (12.3)	9,752 (11.8)	0.070
$PM_{2.5}$	9.93 ± 1.02	9.92 ± 1.00	9.98 ± 1.02	9.99 ± 1.00	0.044
HDL	1.62 ± 0.38	1.41 ± 0.31	1.51 ± 1.07	1.36 ± 0.30	0.167
LDL	3.39 ± 0.78	3.73 ± 0.90	3.42 ± 0.81	3.80 ± 0.92	0.195
TC	5.54 ± 1.04	5.88 ± 1.19	5.49 ± 1.06	5.94 ± 1.22	0.251

* Continuous variables are presented as mean ± standard deviation, while categorical variables are expressed as frequency (%).

Table S5. Sensitivity analysis of the associations of TyG on COPD risk.

Excluding factors	TyG, HR (95% CI)*					*P* for trend
	Q1 (< 6.63)	Q2 (6.63-)	Q3 (6.95-)	Q4 (7.24-)	Q5 (≥ 7.59)	
Excluding COPD cases during first 2 years of follow-up (n=785)						
No. of cases	1,226	1,687	1,904	2,202	2,711	
HR (95% CI)	ref	1.12 (1.04-1.21)	1.12 (1.05-1.22)	1.18 (1.08-1.27)	1.24 (1.14-1.36)	0.004
Excluding participants with poor self-rated health (n=15,160)						
No. of cases	1,169	1,578	1,807/74,391	2,023/73,843	2,360/71,888	
HR (95% CI)	ref	1.10 (1.02-1.19)	1.12 (1.03-1.21)	1.15 (1.06-1.25)	1.21 (1.11-1.33)	< 0.001
Excluding CRP > 10mg/L (n=15,374)						
No. of cases	1,206	1,639	1,906	2,131	2,651	
HR (95% CI)	ref	1.11 (1.03-1.20)	1.13 (1.05-1.22)	1.16 (1.17-1.26)	1.24 (1.13-1.36)	< 0.001

* Adjusted for age, sex, race, Townsend deprivation index, college or university degree, physical activity (moderate MET-hours/week), smoking status, passive smoking, alcohol intake frequency, baseline asthma, high-risk occupation for COPD, $PM_{2.5}$, vegetable intake, fresh fruit intake, oily fish intake, family history of respiratory diseases, baseline diabetes, BMI, HDL, LDL, and TC.

Table S6. Sensitivity analysis of the joint associations of TyG and CRP on COPD risk.

	TyG*CRP, HR (95% CI) *			
	TyG < 7.14 & CRP < 1.88mg/L	TyG < 7.14 & CRP ≥ 1.88mg/L	TyG ≥ 7.14 & CRP < 1.88mg/L	TyG ≥ 7.14 & CRP ≥ 1.88mg/L
Excluding COPD cases during first 2 years of follow-up (n=785)				
No. of cases	2,097	2,051	1,996	3,586
HR (95% CI)	ref	1.61 (1.51-1.72)	1.09 (1.01-1.16)	1.70 (1.59-1.82)
Excluding participants with poor self-rated health (n=15,160)				
No. of cases	2,029	1,890	1,853	3,165
HR (95% CI)	ref	1.60 (1.50-1.71)	1.07 (0.99-1.15)	1.69 (1.58-1.81)
Excluding CRP > 10mg/L (n=15,374)				
No. of cases	2,231	1,855	2,143	3,304
HR (95% CI)	ref	1.55 (1.46-1.66)	1.09 (1.02-1.17)	1.66 (1.55-1.77)

* TyG and CRP were categorized by cut-off values, and combined into a joint variable.

Adjusted for age, sex, race, Townsend deprivation index, college or university degree, physical activity (moderate MET-hours/week), smoking status, passive smoking, alcohol intake frequency, baseline asthma, high-risk occupation for COPD, $PM_{2.5}$, vegetable intake, fresh fruit intake, oily fish intake, family history of respiratory diseases, baseline diabetes, BMI, HDL, LDL, and TC.

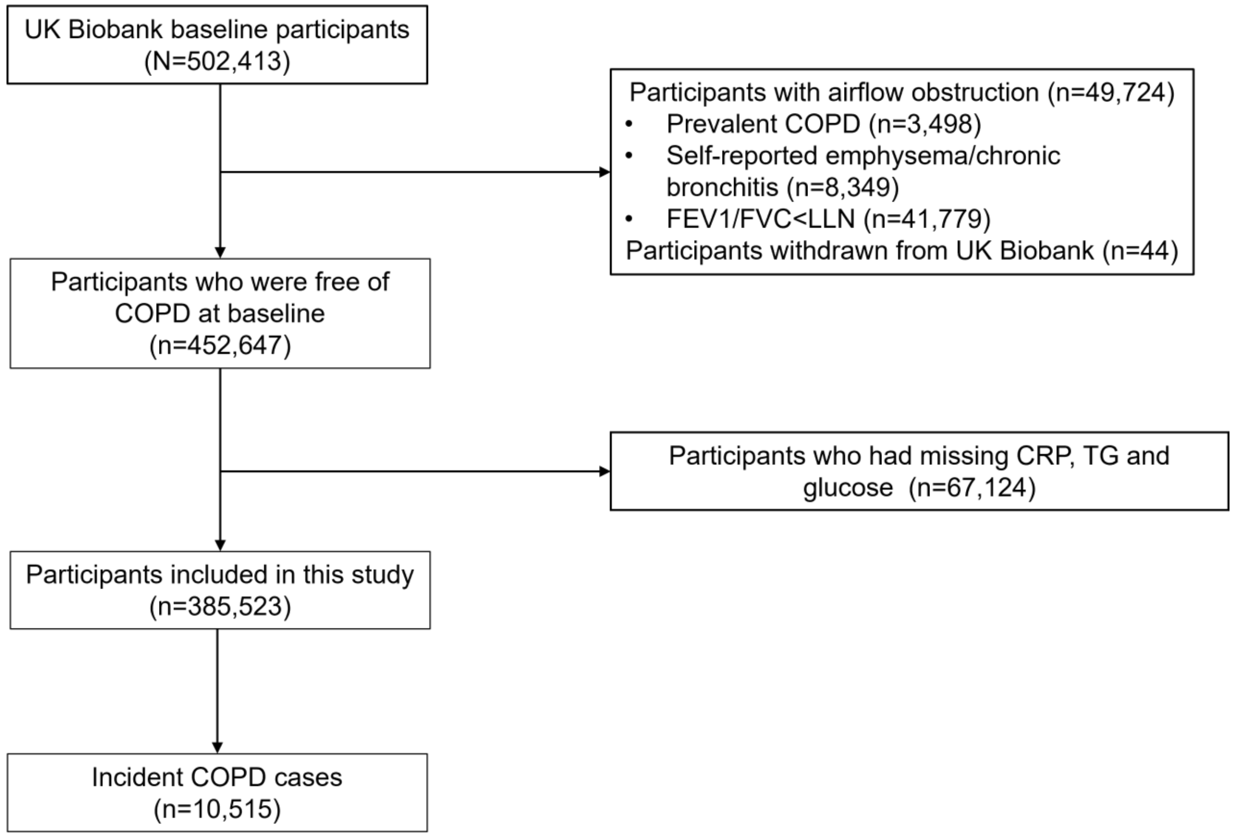

Figure S1. The flow chart for inclusion and exclusion of all participants in this study.

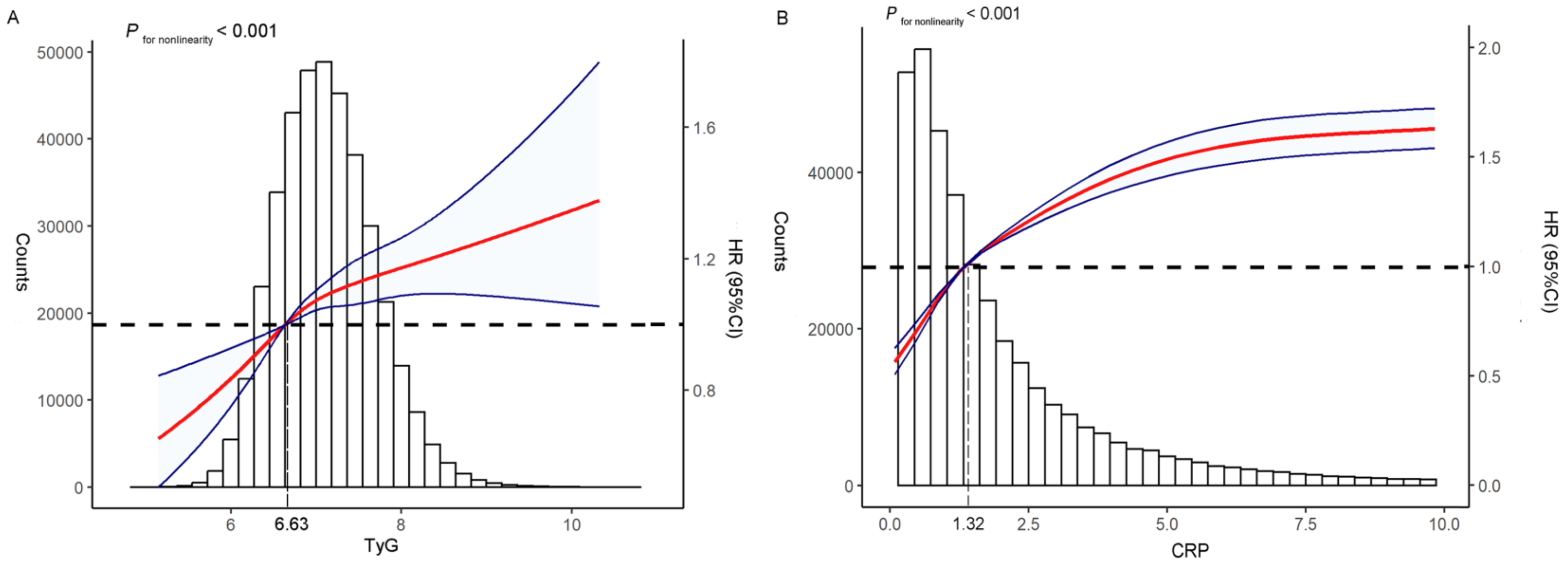

Figure S2. The nonlinear relationship between TyG, CRP and COPD risk. The nonlinear curve was calculated by restricted cubic spline (RCS), and adjusted for baseline age, sex, race, TyG, Townsend deprivation index, college or university degree, physical activity (moderate MET-hours/week), smoking status, passive smoking, alcohol intake frequency, baseline asthma, high-risk occupation for COPD, $PM_{2.5}$, vegetable intake, fresh fruit intake, oily fish intake, family history of respiratory diseases, baseline diabetes, BMI, HDL, LDL, and TC.

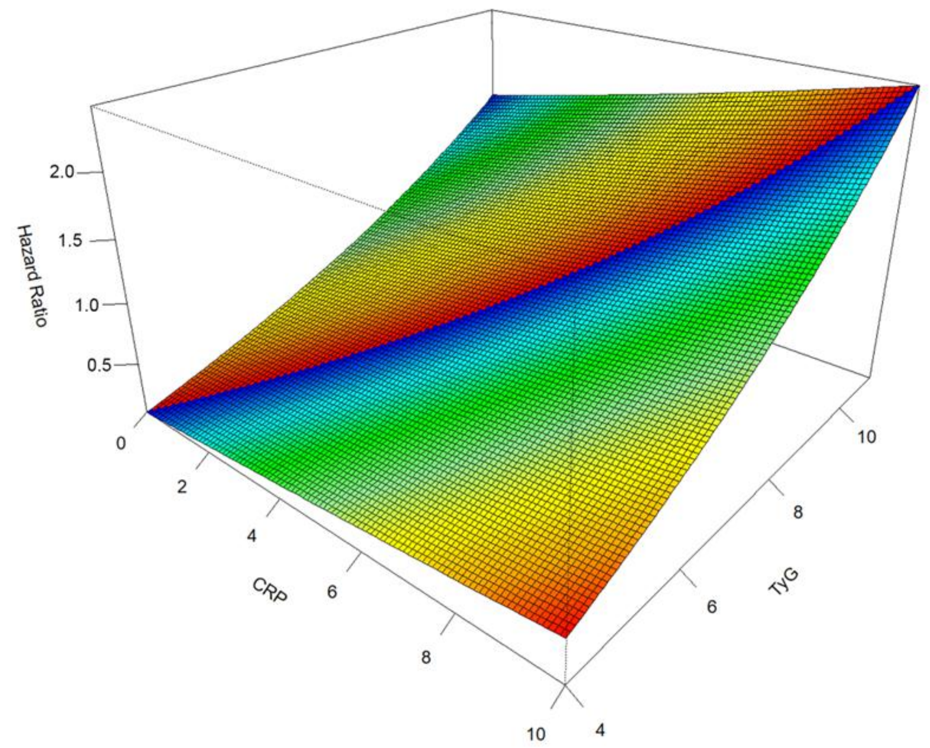

Figure S3. The 3D-surface plot for the nonlinear associations for TyG and CRP on COPD risk. The model was adjusted for baseline age, sex, race, Townsend deprivation index, college or university degree, physical activity (moderate MET-hours/week), smoking status, passive smoking, alcohol intake frequency, baseline asthma, high-risk occupation for COPD, $PM_{2.5}$, vegetable intake, fresh fruit intake, oily fish intake, family history of respiratory diseases, baseline diabetes, BMI, HDL, LDL, and TC.

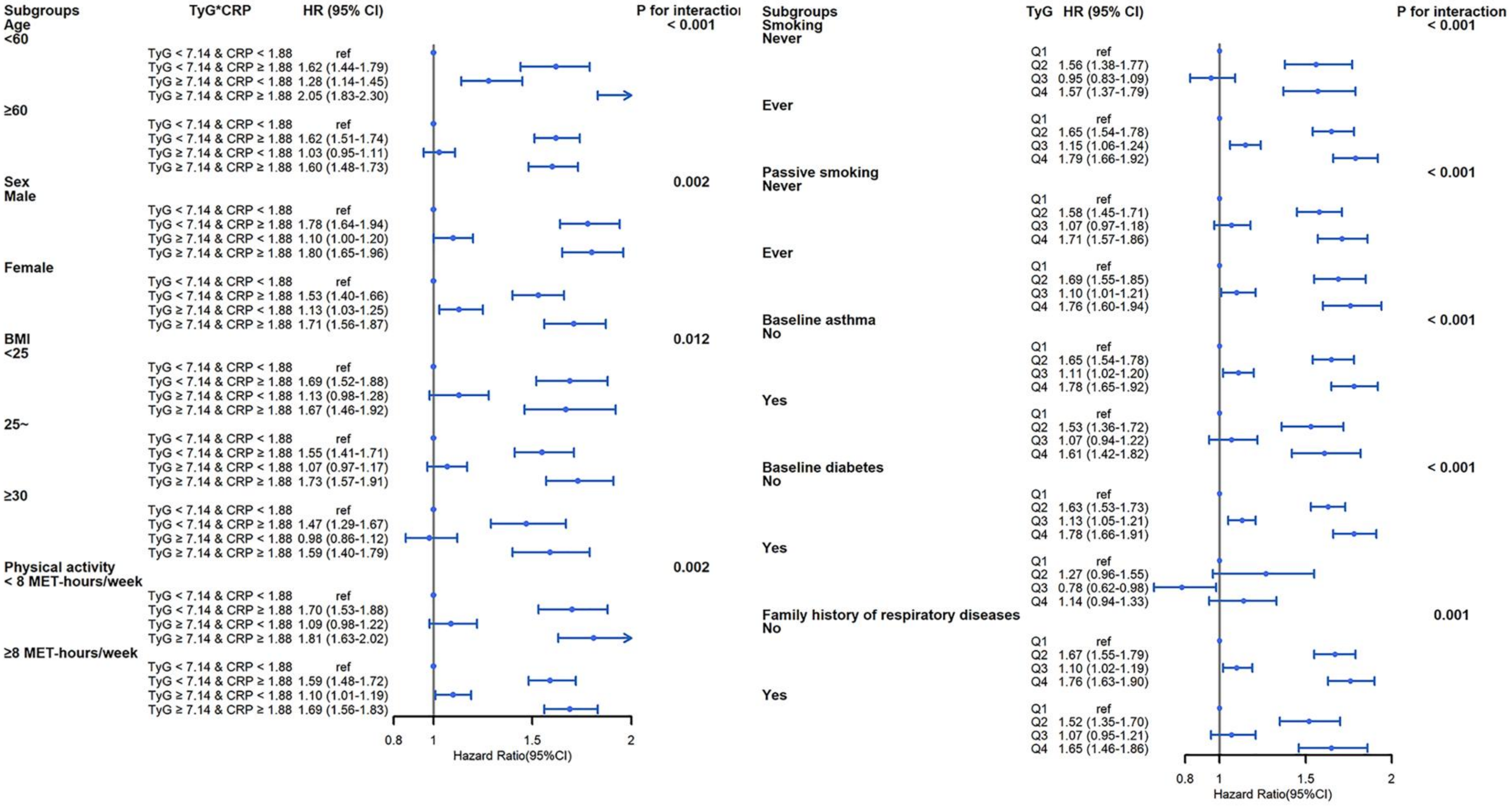

Figure S4. Stratified analysis of the association between TyG and CRP with the risk of COPD.

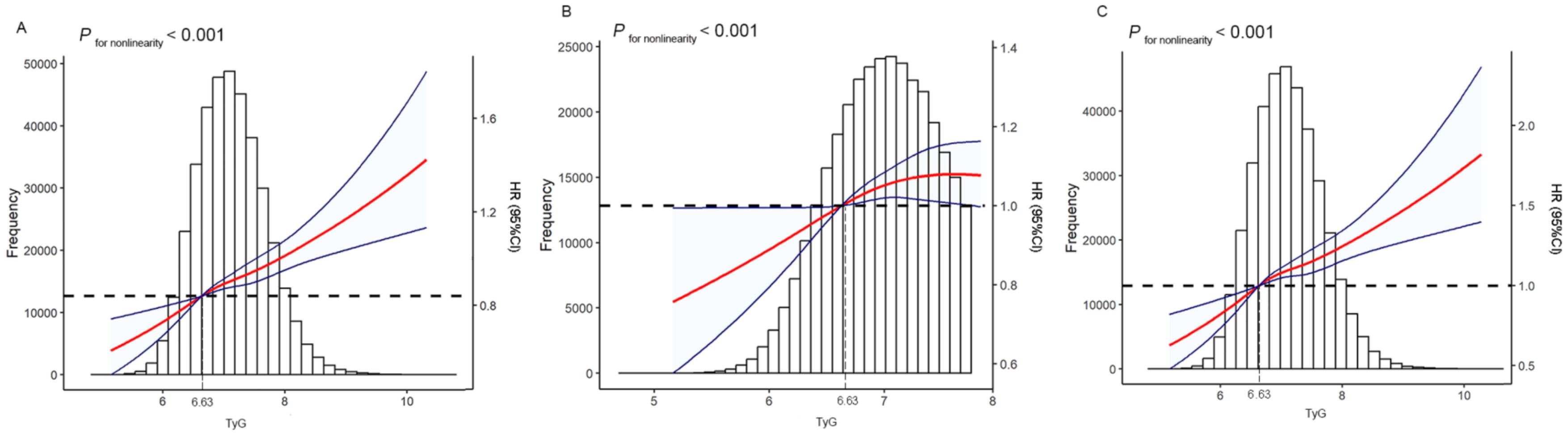

Figure S5. The sensitivity analysis for the nonlinear relationships between TyG and COPD risk. (A) Excluding COPD cases during first 2 years of follow-up (n=785). (B) Excluding participants with poor self-rated health (n=15,160). (C) Excluding CRP > 10mg/L (n=15,374). The three models were adjusted for baseline age, sex, race, CRP, Townsend deprivation index, college or university degree, physical activity (moderate MET-hours/week), smoking status, passive smoking, alcohol intake frequency, baseline asthma, high-risk occupation for COPD, $PM_{2.5}$, vegetable intake, fresh fruit intake, oily fish intake, family history of respiratory diseases, baseline diabetes, BMI, HDL, LDL, and TC.

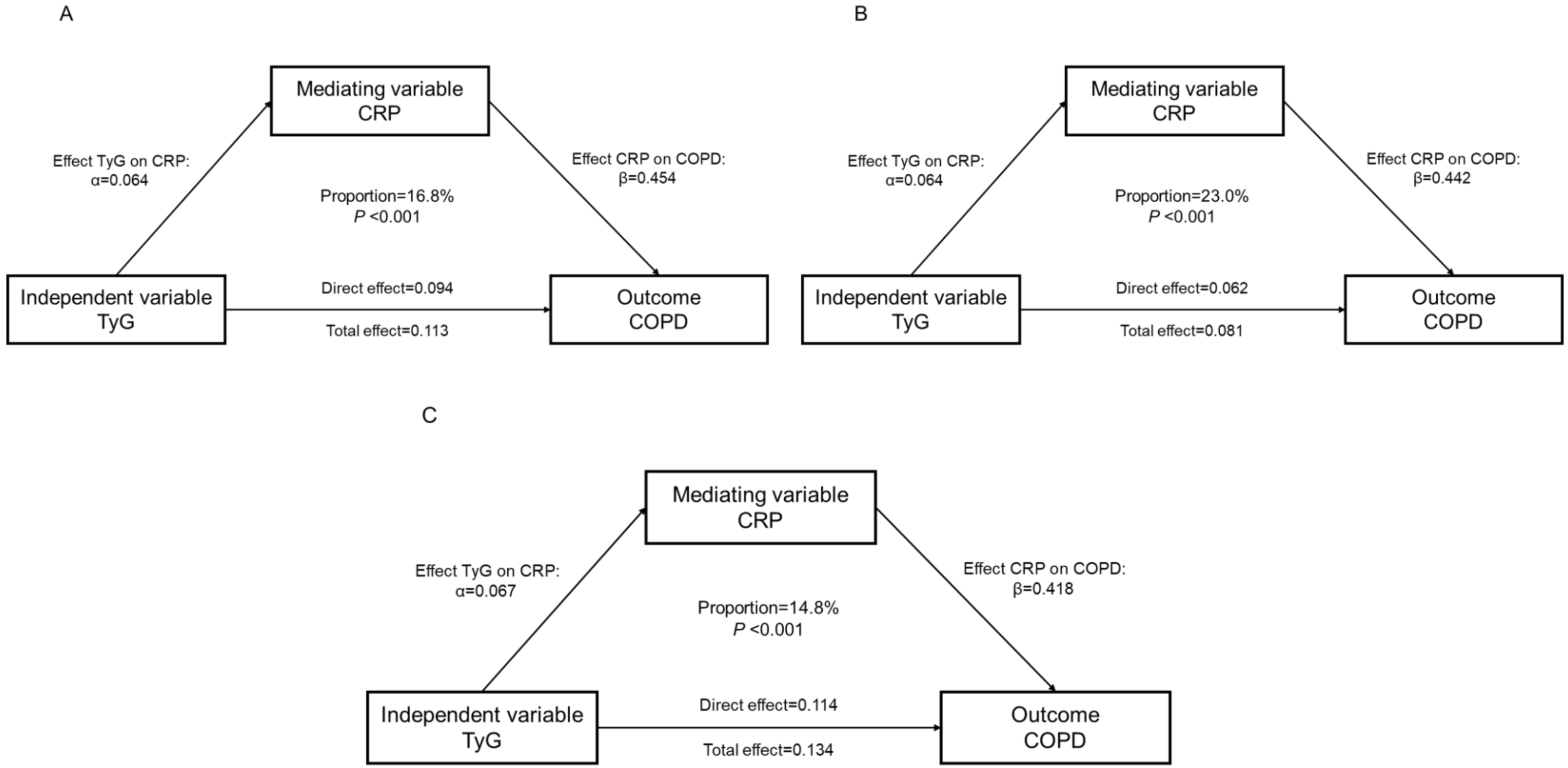

Figure S6. Sensitivity analysis for mediation effect between TyG and CRP on COPD risk. (A) Excluding COPD cases during first 2 years of follow-up (n=785). (B) Excluding participants with poor self-rated health (n=15,160). (C) Excluding CRP > 10mg/L (n=15,374). The mediation analysis models were adjusted for baseline age, sex, race, Townsend deprivation index, college or university degree, physical activity (moderate MET-hours/week), smoking status, passive smoking, alcohol intake frequency, baseline asthma, high-risk occupation for COPD, $PM_{2.5}$, vegetable intake, fresh fruit intake, oily fish intake, family history of respiratory diseases, baseline diabetes, BMI, HDL, LDL, and TC.

- -

Manuscript.docx

Ley Academy

Document Details

Submission ID

trn:oid:::3618:103711085

Submission Date

Jul 7, 2025, 5:17 PM GMT+8

Download Date

Jul 7, 2025, 5:20 PM GMT+8

File Name

20250707171651A149.docx

File Size

160.0 KB

27 Pages

6,099 Words

34,401 Characters

14% Overall Similarity

The combined total of all matches, including overlapping sources, for each database.

Filtered from the Report

- Bibliography
- Quoted Text

Match Groups

57 Not Cited or Quoted 12%
Matches with neither in-text citation nor quotation marks

12 Missing Quotations 2%
Matches that are still very similar to source material

0 Missing Citation 0%
Matches that have quotation marks, but no in-text citation

0 Cited and Quoted 0%
Matches with in-text citation present, but no quotation marks

Top Sources

12% Internet sources
12% Publications
0% Submitted works (Student Papers)

Integrity Flags

0 Integrity Flags for Review

No suspicious text manipulations found.

Our system's algorithms look deeply at a document for any inconsistencies that would set it apart from a normal submission. If we notice something strange, we flag it for you to review.

A Flag is not necessarily an indicator of a problem. However, we'd recommend you focus your attention there for further review.

Match Groups

- **57** Not Cited or Quoted 12%
  Matches with neither in-text citation nor quotation marks
- **12** Missing Quotations 2%
  Matches that are still very similar to source material
- **0** Missing Citation 0%
  Matches that have quotation marks, but no in-text citation
- **0** Cited and Quoted 0%
  Matches with in-text citation present, but no quotation marks

Top Sources

12% Internet sources
12% Publications
0% Submitted works (Student Papers)

Top Sources

The sources with the highest number of matches within the submission. Overlapping sources will not be displayed.

#	Type	Source	%
1	Internet	bmjopenrespres.bmj.com	2%
2	Internet	www.mdpi.com	1%
3	Internet	www.frontiersin.org	<1%
4	Internet	www.researchsquare.com	<1%
5	Internet	bmcmedicine.biomedcentral.com	<1%
6	Internet	journals.lww.com	<1%
7	Internet	pure.eur.nl	<1%
8	Publication	Zhangyu Lin, Jining He, Chenxi Song, Sheng Yuan, Yanjun Song, Xiaohui Bian, Kef...	<1%
9	Internet	www.nature.com	<1%
10	Internet	epiresearch.org	<1%

11	Internet	pubmed.ncbi.nlm.nih.gov	<1%
12	Internet	cardiab.biomedcentral.com	<1%
13	Internet	onlinelibrary.wiley.com	<1%
14	Internet	www.researchgate.net	<1%
15	Internet	library.med.nyu.edu	<1%
16	Publication	Nika Skoro-Sajer, Christian Gerges, Mario Gerges, Adelheid Panzenböck et al. "Us...	<1%
17	Internet	oaepublish.com	<1%
18	Publication	Yuwen Chen, Wenbin Lian, Lunzhe Wu, An'an Huang, Deliang Zhang, Bingchen Li...	<1%
19	Internet	discovery.researcher.life	<1%
20	Internet	pmc.ncbi.nlm.nih.gov	<1%
21	Internet	res.mdpi.com	<1%
22	Internet	synapse.koreamed.org	<1%
23	Publication	"Abstracts of 52nd EASD Annual Meeting", Diabetologia, 2016	<1%
24	Publication	Wei Wang, Mei Tu, Xiu Ping Qiu, Yan Tong, Xiu Li Guo. "The Interplay of Systemic I...	<1%

25 Publication

Zheng Zhu, Xinglin Wan, Jiannan Liu, Dandan Zhang et al. "Vitamin D status and c... <1%

26 Internet

itea4.org <1%

27 Publication

Yuxin Yan, Liyu Zhou, Rui La, Ming Jiang, Dinghua Jiang, Lixin Huang, Wu Xu, Qian... <1%

28 Publication

Lan Chen, Chongjian Wang, Shiyu Zhang, Shengtao Wei, Jinde Zhao, Zilong Zhang.... <1%

29 Internet

bmcpulmmed.biomedcentral.com <1%

30 Internet

link.springer.com <1%

31 Internet

lipidworld.biomedcentral.com <1%

32 Internet

hdruk.github.io <1%

33 Internet

ijmedph.org <1%

34 Internet

www.omicsdi.org <1%

35 Internet

www.science.gov <1%

36 Publication

G. A. Ferns, L. Forster, A. Stewart-Lee, M. Konneh, J. Nourooz-Zadeh, E. E. Anggard... <1%

37 Publication

Han Han, Ying Wang, Tongtong Li, Chengwu Feng, Catherine Kaliszewski, Yang Su... <1%

38 Publication

Ikramulhaq Patel, JingYan Zhang, YinHe Chai, YuShun Qiao, HongJian Gong, Hui X... <1%

39 Internet

dmsjournal.biomedcentral.com <1%

40 Internet

mdpi-res.com <1%

41 Publication

Arshi, B, M Tohidi, A Derakhshan, S Asgari, F Azizi, and F Hadaegh. "Sex-specific re... <1%

42 Publication

Bingxue Wang, Liying Li, Ying Tang, Xingwu Ran. "Joint association of triglyceride ... <1%

43 Publication

Shu Yang, Zhenwei Wang. "The triglyceride–glucose index is a promising predicto... <1%

44 Publication

Yanjun Song, Kongyong Cui, Min Yang, Chenxi Song, Dong Yin, Qiuting Dong, Yin... <1%

The joint effect of triglyceride-glucose index and C-reactive protein levels on the risk of chronic obstructive pulmonary disease: A prospective cohort study.

Author: Jialiu He[1], Mengxia Li[2], Pengfei Luo[2], Zheng Zhu[2], Jian Su[2,3], Ran Tao[2,3], Jinyi Zhou[2,3], Ming Wu[1,2, *] and Xikang Fan[2, *]

Abstract

Background: The triglyceride-glucose index (TyG) and C-reactive protein (CRP) are key biomarkers on clinical diagnosis, each related to lung dysfunction. However, the relationship of both indexes with the risk of chronic obstructive pulmonary disease (COPD) is still unclear. This study purposes to focus on the individual and joint associations of TyG and CRP levels with COPD risk.

Methods: This cohort study utilized baseline TyG and CRP data from the UK Biobank. Hazard ratios (HRs) and 95% confidence intervals (CIs) for COPD risk associated with TyG and CRP levels were calculated through Cox regression models. Receiver operating characteristic (ROC) curves were conducted to determine the optimal cut-off values for TyG and CRP, which were combined into a joint variable. Kaplan-Meier (KM) method was utilized to analyze cumulative hazard, while joint analysis was employed for evaluating the joint risk. Stratified and sensitivity analyses were also performed to assess the associations within subgroups, and mediation effect of TyG on COPD risk via CRP levels was assessed.

Results: This study enrolled 385,523 individuals, with 10,515 COPD cases were recorded in follow-up. Compared to the lowest quintile, individuals with higher TyG and CRP had increased risk of COPD (all HRs >1.00). The optimal cut-off values of

iThenticate Page 7 of 33 - Integrity Submission Submission ID trn:oid:::3618:103711085

TyG and CRP were 7.14 and 1.88 mg/L, and we found that the simultaneous elevation of both TyG and CRP significantly increased the risk of COPD. Moreover, the joint effect was stronger in participants < 60 years old, males, smokers or passive smokers, those with body mass index (BMI) < 25.0 kg/m^2, and those without baseline diabetes, asthma, or a family history of respiratory diseases ($P_{\text{for interaction}} < 0.05$). Moreover, the effect of TyG on COPD was significantly mediated by CRP, explaining almost 15.6% of this influence.

Conclusions: These results underscored the individual and joint effects of TyG and CRP upon COPD risk, indicating their usefulness as biomarkers for early risk assessment.

Key words: Triglyceride-glucose index, C-creative protein, Chronic obstructive pulmonary disease, Joint effect

Background

As a globally prevalent chronic condition, COPD manifests as persistent airway inflammation and progressive airflow obstruction, contributing significantly to mortality rates and global disease burden[1]. The latest data from the World Health Organization (WHO) revealed that nearly 3.5 million deaths were attributed to COPD in 2021 with a global prevalence of approximately 10.3%, which has undoubtedly imposed a heavy burden on the healthcare systems[2]. The condition’s vague clinical presentation often delays diagnosis until advanced stages. Hence, investigating the underlying causes of COPD is crucial for mitigating its impact on morbidity and

mortality[3]. Despite established associations with cigarette smoking, environmental pollutants, and genetics, the specific causes of COPD are still unclear, and its pathogenesis is not fully explained[4]. This uncertainty highlights the need for more work exploring possible contributions.

In recent years, studies have shown that metabolic disturbances may promote the development of COPD, further expanding understanding of its etiological factors[5, 6]. Clinical evidence indicates that abnormalities in glucose metabolism are not only common in COPD patients, but also associated with worsening of lung tissue function[7]. Insulin resistance has increasingly been recognized as a key feature linking metabolic disturbances to chronic diseases[8]. Recent studies have identified the triglyceride-glucose index (TyG) as a recognized indicator of metabolic or cardiovascular diseases via routine clinical evaluation[9]. Emerging evidence suggests that TyG shows diagnostic potential during preclinical respiratory assessments. According to a recent observational study, increased TyG levels are linked to a greater incidence of exertional dyspnea, and a positive relationship with lung dysfunction[10]. Another cross-sectional study briefly analyzed the distribution of TyG across sex and its association with COPD[11]. However, existing studies have focused mainly on the impact of TyG with lung function, while evidence from large-scale cohort studies remains limited. Consequently, the investigation of TyG as a potential risk factor may give new insights into the pathogenesis of COPD.

Furthermore, chronic inflammation induced by insulin resistance is also believed to be a factor in lung tissue damage and remodeling, which leads to an abnormal decline

in lung function[12]. Inflammation is thought to underlie lung tissue remodeling and worsening airflow obstruction in individuals with COPD[13]. As a commonly examined biomarker of systemic inflammation, higher CRP levels are related to greater risk of developing COPD, as well as worse clinical outcomes such as exacerbations and mortality[14, 15]. Higher levels of CRP are frequently observed in those diagnosed with COPD, underscoring inflammatory component of the disease[15, 16].

Moreover, the elevation of TyG is often associated with increased inflammatory responses, including higher CRP level. Because of their respective roles as risk factors for chronic diseases, researchers have recently started to investigate how TyG and CRP interact to increase the potential risk[17]. These effect of higher TyG and CRP levels has already been discussed in cardiovascular diseases[18]; however, an in-depth exploration of their effects is still lacking in COPD. Therefore, this study aims to explore the independent and joint effects of TyG and CRP for COPD risk using long-term cohort data from the UK Biobank. By investigating these associations, we hope to identify novel indicators that could facilitate the early detection of COPD, thereby establish a theoretical basis for its prevention efforts.

Methods

Study population

Data were derived from the UK Biobank, a longitudinal cohort in the United Kingdom with over half a million participants. At baseline, participants aged 40-69 years from 22 research centers were recruited from 2006 to 2010[19]. During recruitment, participants

underwent an in-person assessment that included a touchscreen questionnaire, physical examinations, and blood sample collection by trained nurses.

In this study, people with airway obstruction were excluded initially[19], encompassing individuals with clinical diagnosed COPD[20], participants who indicated a history of emphysema or chronic bronchitis[20], and those with the ratio of forced vital capacity (FVC) to forced expiratory volume in 1 second (FEV_1) less than the lower limit of normal (LLN) [21]. Subsequently, we also excluded individuals whose triglyceride (TG), fasting blood glucose (FBG), and CRP levels were not measured (n=67,124). Finally, 385,523 participants were included at baseline (Figure S1).

Measurement of TyG and CRP

The formula for calculating TyG was as follows[22, 23]:

$$TyG = \ln(TG\ (mg/dL) \times FBG\ (mg/dL)\ /2).$$

Baseline TG and FBG were measured by glycerol-3-phosphate oxidase-peroxidase analysis and hexokinase analysis on automatic biochemical analyzer Beckman Coulter AU5800. The units of measurement were mmol/L, which were converted to mg/dL. Besides, CRP was measured by immunoturbidimetric-high sensitivity analysis, with a detection range of 0.08-80.00 mg/L.

Definition of outcome

New-onset COPD cases were identified by linking data with the National Health

Service (NHS) health care registries, utilizing censoring dates of 30 September 2021 for England, 31 July 2021 for Scotland, and 28 February 2018 for Wales. Data regarding the date and causes of death subsequent to COPD diagnosis were obtained from national death registries, with availability up to 30 September 2021 for England and Wales, and through 31 October in Scotland. Diagnostic information was classified using the 10th edition of the International Classification of Diseases (ICD-10), with COPD defined by codes J40-J44[20].

Covariate ascertainment

Covariates were collected from baseline database, including demographic characteristics (age, sex, race, and socioeconomic status), daily behaviors (smoking, drinking, passive smoking, physical activity, and nutritional habits), environmental exposure (including $PM_{2.5}$, high-risk occupation for COPD), personal history of asthma and diabetes, and family history of respiratory diseases. Moreover, lipid biochemical indexes were included in the analysis. Total cholesterol (TC, mmol/L) levels were measured by using the cholesterol oxidase-peroxidase enzymatic colorimetric method. Low-density lipoprotein (LDL) and high-density lipoprotein (HDL) were both quantified through enzymatic methods and are expressed in mmol/L.

In detail, body mass index (BMI, kg/m^2) was defined as weight (kg) divided by the square of height in meters. Physical activity level was quantified as metabolic equivalent task hours per week (MET-hours/week) for moderate activity[24]. $PM_{2.5}$ exposure was evaluated for each center using land use regression utilizing 2010 data;

meanwhile, high-risk occupations for COPD were selected by participants from predefined options in the questionnaire[19]. Baseline asthma was defined according to self-reported information and hospital admissions marked with J45-J46. Moreover, diabetes was defined as follows: (1) diagnosed before recruitment; (2) self-reported use of diabetes medications; (3) registered in the hospital records as diabetic; or (4) hemoglobin A1c (HbA1c) levels ≥ 6.5%[25]. The diagnosed diabetes was coded as E10-E14.

Statistical analysis

Person-years were accrued starting from the assessment date, with follow-up censored at whichever came first: being diagnosed with COPD, passing away, becoming lost to follow-up, or reaching the deadline of this study. Continuous variables are reported as the means ± standard deviations (SDs), and categorical variables are presented as frequencies and percentages (n, %). Statistical differences between participants with and without TyG and CRP data were evaluated by calculating the standardized mean difference (SMD), where values over 0.2 were interpreted as meaningful differences.

Besides, hazard ratios (HRs) and 95% confidence intervals (CIs) for COPD risk were derived from Cox proportional hazard models. Three models were developed to estimate the associations of TyG and CRP with COPD risk: (1) Model 1 was adjusted for age, sex, and race; (2) Model 2 plus adjusted for socioeconomic status, daily lifestyles, environmental exposure, baseline asthma, and family history of respiratory diseases; and (3) Model 3 further included BMI, baseline diabetes, and biochemistry

indexes (TC, HDL, and LDL). We divided TyG and CRP as quintiles (Q1 to Q5), and defined the lowest quintile as the reference to show the associations. Besides, restricted cubic spline (RCS) curves and 3D-surface plots were generated to visualize the dose-response relationships for TyG and CRP on COPD risk. The list of covariates incorporated in the RCS was consistent with that in Model 3.

To analyze the joint effect of TyG and CRP, we first performed ROC curves to determine the optimal cut-off values for both variables based on their area under the curves (AUC). TyG and CRP were categorized into high and low groups (< cut-off, ≥ cut-off), and a four-level categorical variable was constructed according to their optimal cut-off values identified through ROC curves: (1) low TyG and low CRP (defined as the reference); (2) low TyG and high CRP; (3) high TyG and low CRP; and (4) high TyG and high CRP. Meanwhile, Kaplan-Meier (KM) curves were plotted to illustrate the cumulative hazard across TyG-CRP joint categories, and the log-rank test was used to examine group-level differences on COPD risk. To estimate the magnitude of the joint effect, we subsequently constructed Cox models adjusted for the complete set of covariates specified in Model 3.

Stratified analysis was conducted to evaluate the association between the joint TyG-CRP and COPD risk across different subgroups. Specifically, multiplicative interaction terms between the joint variable and subgroup variables (e.g., age, sex, smoking, passive smoking, BMI, physical activity, baseline asthma, baseline diabetes, and family history of respiratory diseases) were included in Cox models. The likelihood ratio tests were conducted to compare models with and without interaction terms to

assess the significance to effect modification. Moreover, the Baron & Kenny method was applied to fit the mediation and outcome models, to evaluate both the direct effect of TyG on COPD risk and its indirect effect mediated by CRP. Meanwhile, the potential confounders in Cox models were also adjusted in the mediation analysis.

Finally, sensitivity analysis was performed to validate the robustness of results, which was performed as follows: (1) excluding individuals who developed COPD during the first 2 years of follow-up; (2) excluding people self-reporting poor health status; and (3) excluding those with extremely high CRP levels (> 10 mg/L). All analyses were performed by using R software (version 4.4) and SAS software (version 9.4). Statistical significance was determined using two-sided tests, with a threshold of $P < 0.05$.

Results

Baseline characteristics

No notable differences were found between included and excluded participants, as all SMDs < 0.2 (Table S1). Over a median follow-up period of 12.28 years, a total of 10,515 COPD cases were diagnosed. The average age was 56.5 years at baseline, with females comprising almost 54.2%. We listed baseline characteristics categorized by TyG, and found the proportion of females were decreased from Q1 (< 6.63) to Q5 (≥ 7.59), whereas BMI increased with higher TyG level. Participants with increased TyG had higher CRP levels, proportion of asthma, and tended to frequent smoking. The proportion of tertiary educated and frequency of physical activity was lower among

participants, and a total of 23,050 people had baseline diabetes (6.0%).

After classifying participants into five groups based on quintiles of CRP, we observed that the proportions of baseline diabetes, asthma, and family history of respiratory diseases increased among individuals with higher CRP level (Table S2). Furthermore, we compared the baseline characteristics between participants excluded solely due to $FEV_1/FVC < LLN$ and those excluded based on clinical diagnosis or self-reported respiratory disease among individuals with baseline airway obstruction. Apart from differences in alcohol consumption frequency and a family history of respiratory diseases, no significant disparities were observed (Table S3).

Associations of TyG, CRP with COPD risk

Among the total participants, we observed that comparing with the lowest quintile (Q1), higher TyG levels were associated with elevated COPD risk in each model ($P_{\text{for trend}} <$ 0.001). Upon adjustment for covariates, the risk also increased by approximately 25% when TyG ≥ 7.59 (HR=1.25, 95% CI: 1.15-1.36). Besides, higher risk was also observed with higher CRP levels in Model 3 (Q2 vs. Q1: HR=1.22, 95% CI: 1.12-1.33; Q3 vs. Q1: HR=1.48, 95% CI: 1.37-1.61; Q4 vs. Q1: HR=1.79, 95% CI: 1.66-1.94; Q5 vs. Q1: HR=2.28, 95% CI: 2.11-2.46, respectively).

Moreover, the nonlinear trends of TyG and CRP with COPD risk were illustrated in RCS plots (Figure S2). Both TyG and CRP exhibited a nonlinear positive association with COPD risk, with all $P_{\text{for nonlinearity}} < 0.001$. When considering TyG and CRP simultaneously, the 3D-surface also showed an increasing trend in both higher levels

(Figure S3).

Joint effect analysis

To evaluate the joint effect, we firstly categorized TyG and CRP based on their optimal cut-off values. As shown in Figure 1, the cut-off values were 7.14 and 1.88mg/L for TyG and CRP, with the AUC values of 0.579 and 0.648, respectively. We constructed a four-category joint variable derived from the cut-off of TyG (< 7.14, ≥ 7.14) and CRP (< 1.88 mg/L, ≥ 1.88 mg/L), to assess their combined effect on COPD risk. When we combined TyG and CRP, we found baseline characteristics of subgroups were similar, as most of SMDs < 0.2 (Table S4).

Over the follow-up period, the cumulative hazard of COPD differed significantly across the TyG-CRP joint exposure groups ($P_{\text{for log-rank}} < 0.05$). Compared with reference group, the subgroup with higher TyG (≥ 7.14) and low CRP (< 1.88 mg/L) showed a modest increase in cumulative hazard (Table 2). In contrast, individuals with higher CRP and TyG < 7.14 exhibited a more pronounced increase, suggesting a stronger effect of CRP levels. Besides, the highest cumulative hazard was observed in higher levels of both TyG and CRP. Furthermore, a significant increasing trend in COPD risk was observed as both TyG and CRP levels increased by adjusting all covariates in Cox regression model. In details, the highest risk (HR=1.74, 95% CI: 1.63-1.85) was observed among the group of CRP ≥ 1.88 mg/L and TyG ≥ 7.14 (Figure 3).

Stratified and sensitivity analysis

Within stratified analysis, the association between the joint TyG-CRP variable and COPD risk varied across different subgroups. For participants aged younger than 60 years, the joint HR for COPD risk was higher than those who aged ≥ 60 years ($P_{\text{for interaction}} < 0.001$). A more pronounced effect modification of the association was also observed among current smokers and passive smokers, and the risk was also more significant in individuals with BMI < 25.0 kg/m^2 (all $P_{\text{for interaction}} < 0.05$). In addition, among participants with low levels of physical activity (< 8 MET-hours/week), the joint risk associated with elevated CRP (≥ 1.88mg/L) and TyG (>7.14) was higher compared to those with higher activity levels ($P_{\text{for interaction}}$ =0.002). Notably, the joint effect was more significant in those without baseline diabetes, asthma, or family history of respiratory diseases (Figure S4).

As shown in Table S5, TyG remained significantly positive association with COPD risk after excluding various subgroups in sensitivity analysis (all $P_{\text{for trend}} < 0.05$). Nonlinear dose-response curves also illustrated the positive associations between TyG and COPD risk (Figure S5). Moreover, we observed that regardless of TyG level, higher CRP could significantly increase COPD risk (Table S6).

Mediation analysis

Figure 4 summarized the mediation effects linking TyG and CRP to COPD risk. Higher level of CRP mediated approximately 15.6% of association between TyG and COPD risk ($P < 0.001$), with the direct effect coefficient was 0.012. In the sensitivity analysis (Figure S6), the mediation effect was slightly lower in individuals without abnormal

CRP levels (14.8%) and higher in baseline healthy participants (23.0%).

Discussion

While the role of TyG as a metabolic marker is well established, its linkage to inflammation has garnered increasing attention. As a representative inflammatory biomarker, CRP provides further insights into this relationship[26]. However, their combined effect on COPD risk has not yet been investigated. Our findings extend existing evidence by demonstrating that the combined elevation of TyG and CRP significantly increases COPD risk. Notably, we demonstrated the joint effect of both indexes, with the highest risk observed when both markers were elevated. Furthermore, our results indicated a mediation relationship between TyG and CRP with COPD risk.

The association between metabolic indicators and chronic lung diseases is increasingly being explored. The main advantage of TyG as a metabolic marker is the capacity to jointly indicate insulin resistance and lipid metabolism, which helps to capture the characteristics of metabolic dysfunction. Liu et al. reported higher TyG levels were positively associated with increased risk of acute myocardial infarction and sudden cardiac arrest among Chinese[27]. In 2022, a Swedish follow-up study identified elevated TyG as a novel biomarker for predicting COPD risk, reporting a HR of 1.72 (95% CI: 1.41-2.09)[28]. Our results also confirmed that as TyG levels increased, the risk of COPD consistently raised, providing new evidence for the relationship between TyG as a metabolic indicator and COPD risk.

The exacerbation of airway inflammation may initially stem from disruptions in

insulin signaling pathways. Insulin resistance enhances oxidative stress and activates inflammatory pathways (NF-κB and MAPK pathway) in turn, exacerbating airway inflammation[29]. Meanwhile, insulin resistance impairs normal immune cell function in the lungs and dysregulated immunity, thus aggravating airway inflammation and tissue damage[30]. Recently, Ruan et al. highlighted the insulin metabolic network is involved in airway inflammation, and poor prognosis of COPD[31]. Ferreira et al. demonstrated in diabetic mice that insulin could influence airway alterations through its effects on immune regulation[32]. These mechanisms proposed how insulin resistance implicates in COPD pathogenesis, thus stressing the relevance of correction of metabolic disturbances as a prospective strategy for COPD prevention and management.

On the other hand, CRP further facilitates to pulmonary dysfunction by mediating the migration and functional stimulation of innate immune cells (such as neutrophils or macrophages)[33]. These activated phagocytes secrete matrix-degrading proteases and inflammatory mediators that collectively induce alveolar epithelial injury, compromise respiratory membrane integrity, and ultimately lead to bronchoconstriction[34]. Furthermore, inflammation can induce oxidative stress, which exacerbates lung function decline and promotes the transcription of additional inflammatory mediators, thereby perpetuating the vicious cycle of inflammation and oxidative damage[35]. The combined assessment of the both indexes has already been applied in the risk prediction and prognosis of cardiovascular diseases and cancers, but its impact on COPD risk still requires further evidence. We identified a significant association on the joint effect, with

a similar trend observed on other diseases from previous study[36]. This also highlights the universality of the combined index for estimating risk of diseases, which can aid in the early warning and identification of COPD, providing important evidence for stratified screening and management of high-risk populations.

In this study, we utilized cut-off values to categorize TyG and CRP levels, which allowed for a more clinically relevant stratification of risk. Currently, most studies focused on adverse cardiovascular events like atherosclerosis and aortic stenosis[17, 37], with lack of evidence on COPD risk. Additionally, the optimal cut-off values of TyG vary significantly across different diseases, and some researchers use the median or mean of TyG as the basis for grouping, resulting in a lack of a unified and clear standard[36]. However, despite the lower AUC value in this study, TyG still showed potential in identifying individuals at risk of COPD. On the other hand, while the critical value of CRP is well-documented in acute respiratory diseases, its optimal cut-off for COPD onset remains controversial. According to previous meta-analysis in 2017, CRP had often been associated with acute exacerbations or mortality among COPD patients, leading to higher cut-off value such as 3 mg/L, to characterize poor prognosis[15]. The cut-off values determined using the ROC curve can more effectively differentiate individuals with different risk levels, providing more targeted reference for the early detection of COPD and clinical decision-making. Future studies are need to combine clinical features and large-scale population data to validate its clinical applicability.

In terms of stratified analysis, we observed that the combined risk of TyG and CRP for COPD was more significant among smokers or passive smokers. It is well-known

that smokers increase oxidative stress and inflammation and damage airways which can cause impaired lung function. Metabolic disorders are one of the major consequences of smoking[38]. Interestingly, the joint effect appeared stronger among individuals without asthma or a family history of respiratory disease. This may be explained by the reduced interference from genetic or existing conditions in those without such history, which allows the metabolic and inflammatory effects to emerge more prominently. Meanwhile, we also observed an opposite trend when CRP < 1.88 mg/L and TyG ≥ 7.14 among baseline diabetes patients. Diabetes cases often already have insulin resistance[39], which may eliminate the effect of TyG. On the other hand, participants with baseline diabetes may be receiving medication (such as insulin injection or hypoglycemic drugs), which can alter the impact of TyG and CRP levels on COPD risk. When CRP is lower, indicating the absence of significant inflammation, a protective effect may emerge, further reducing COPD risk. In addition, the combined effect was more significant among males, and people younger than 60 years old, and those with lower BMI. Due to higher smoking rate in males, the impact of TyG and CRP on COPD risk may be more pronounced, leading to stronger effect compared to females[40]. Among those who are younger and have lower BMI, the physiological response to metabolic and inflammatory disturbances may be more pronounced, thereby amplifying their risk of COPD[41].

These results point to a possible mediating pathway involving CRP in the link between TyG and COPD risk. Li et al. explored the mediating roles of TyG and CRP in the association between obesity and colorectal cancer risk in 93,659 participants,

iThenticate Page 23 of 33 - Integrity Submission Submission ID trn:oid:::3618:103711085

confirming that both CRP and TyG independently or synergistically increase the risk[42]. Chen et al. have found that CRP has also been proposed to mediate the association between TyG levels and lung function[43]. However, we did not proceed with an additional mediation analysis involving TyG as a potential intermediate factor linking CRP to COPD risk, given that TyG reflects long-term metabolic status, whereas inflammation is a more immediate clinical response. Therefore, we believe that it is more plausible for changes in metabolic characteristics to mediate COPD onset through inflammation. In the sensitivity analysis, the results remained stable, which indirectly supports the rationale of using CRP as a mediator in the pathway through which TyG contributes to COPD.

Strengths and limitations

There were several strengths of this study. First, it utilized data from the UK Biobank, providing a robust basis for the findings. The follow-up was long term, which allowed for correct assessment of the associations of baseline TyG and CRP on COPD risk, which brought important insights into possible causal relationships. Moreover, this study extended previous evidence by demonstrating a joint impact of TyG and CRP on COPD, while mediation analysis suggested that CRP partially mediates for the association.

Notwithstanding the above advantages, some limitations could not be avoided. The potential biases and confounding factors cannot be eliminated completely, for the causal conclusions still remained uncertain. Second, we used baseline measurement data only, and did not consider the possible dynamic effects of TyG and CRP over time, which

iThenticate Page 23 of 33 - Integrity Submission Submission ID trn:oid:::3618:103711085

iThenticate Page 24 of 33 - Integrity Submission Submission ID trn:oid:::3618:103711085

could possibly impede a better understanding of their long-term effects. Moreover, the potential impact of pharmacological interventions, including therapies for diabetes or inflammation, was not taken into account in this analysis. Additionally, the incidence of COPD was identified solely using ICD-10 codes, which carries a risk of misclassification. Future research should include multi-time point studies and trajectory analyses to clarify how TyG and CRP change over time during follow-up. Moreover, the cut-off values for TyG and CRP were determined by ROC curve analysis within the same cohort used for outcome modeling. This approach may lead to overfitting and optimistic estimates of risk prediction. Due to resource limitations, internal validation methods such as bootstrapping or cross-validation were not performed. Although statistically significant, the AUC values suggest limited discriminatory power of TyG and CRP when used individually. Despite adjustment for covariates, potential confounding cannot be entirely ruled out, and the mediation analysis results may still be biased. While the relationship between TyG and CRP with COPD risk was demonstrated, it does not delve deeply into the specific molecular mechanisms, leaving a gap in understanding the precise pathways through which these factors influence the disease. Furthermore, it needs in-depth research for underlying mechanisms to confirm our results.

Conclusions

Overall, this study indicated that both TyG and CRP, whether individually or in combination, were associated with higher COPD risk. Additionally, CRP mediated the

iThenticate Page 24 of 33 - Integrity Submission Submission ID trn:oid:::3618:103711085

association between TyG and COPD risk, indicating a potential link involving metabolic dysfunction and inflammation status. These findings highlight the value of incorporating TyG and CRP as clinical biomarkers for risk stratification of COPD.

Declarations

Ethics approval

The ethics approval was obtained from the North West Multi-Center Research Ethics Committee (06/MRE08/65). The informed consent was provided before investigation.

Competing interest

All authors declared that they have no conflict of interest.

Consent for publication

Not applicable for this study.

Funding

This study was supported by the grant from Key Project of Medical Science Research of Jiangsu Provincial Health Commission (No. K2023072), and Jiangsu Provincial Health Commission Medical Research Project Approval (No. H2023022).

Data Availability

The dataset supporting this study is available from the UK Biobank, though access is

subject to certain restrictions. The dataset was utilized under the study's license and is not openly available. Access can be granted upon formal request and approval by the UK Biobank (https://www.ukbiobank.ac.uk/). This study was accessed the UK Biobank resource under application ID 84525.

Acknowledgements

We acknowledge the contributions of all participants, as well as the support from the UK Biobank team. Thanks to MPH. Mengyao Li at Luoyang Central Hospital affiliated to Zhengzhou University for her help and contributions to coding verification.

Author contributions

XF, MW, and JH performed the design of this study. JH, ML, and PL were responsible for data analysis. ZZ, JS, RT, and JZ were responsible for data interpretation. JH wrote the manuscript. JS, RT, JZ, MW and XF reviewed this study. All authors have already approved the manuscript.

Abbreviation list

TyG: triglyceride-glucose index; ICD-10: the 10th edition of the International Classification of Diseases; CRP: C-reactive protein; MET: metabolic equivalent; FEV_1: forced expiratory volume in the first second; LLN: lower limit of normal; RCS, restricted cubic spline; FVC: forced vital capacity; COPD: chronic obstructive pulmonary disease; Mets: metabolic syndrome; BMI, body mass index.

References

1. Christenson SA, Smith BM, Bafadhel M, Putcha N: Chronic obstructive pulmonary disease. *Lancet* 2022, 399:2227-2242.
2. Collaborators GBDCoD: Global burden of 288 causes of death and life expectancy decomposition in 204 countries and territories and 811 subnational locations, 1990-2021: a systematic analysis for the Global Burden of Disease Study 2021. *Lancet* 2024, 403:2100-2132.
3. MacLeod M, Papi A, Contoli M, Beghe B, Celli BR, Wedzicha JA, Fabbri LM: Chronic obstructive pulmonary disease exacerbation fundamentals: Diagnosis, treatment, prevention and disease impact. *Respirology* 2021, 26:532-551.
4. Venkatesan P: GOLD COPD report: 2024 update. *Lancet Respir Med* 2024, 12:15-16.
5. Kim OH, Lee KN, Han K, Cho IY, Shin DW, Lee SW: Association between metabolic syndrome and chronic obstructive pulmonary disease development in young individuals: a nationwide cohort study. *Respir Res* 2024, 25:414.
6. Luo X, Zeng W, Tang J, Liu W, Yang J, Chen H, Jiang L, Zhou X, Huang J, Zhang S, et al: Multi-modal transcriptomic analysis reveals metabolic dysregulation and immune responses in chronic obstructive pulmonary disease. *Sci Rep* 2024, 14:22699.
7. Yu D, Chen T, Qin R, Cai Y, Jiang Z, Zhao Z, Simmons D: Association between lung capacity and abnormal glucose metabolism: findings from China and

Australia. *Clin Endocrinol (Oxf)* 2016, 85:37-45.

8. Lee SH, Park SY, Choi CS: Insulin Resistance: From Mechanisms to Therapeutic Strategies. *Diabetes Metab J* 2022, 46:15-37.

9. Alizargar J, Bai CH, Hsieh NC, Wu SV: Use of the triglyceride-glucose index (TyG) in cardiovascular disease patients. *Cardiovasc Diabetol* 2020, 19:8.

10. Wu TD, Fawzy A, Brigham E, McCormack MC, Rosas I, Villareal DT, Hanania NA: Association of Triglyceride-Glucose Index and Lung Health: A Population-Based Study. *Chest* 2021, 160:1026-1034.

11. Guo J, Yang J, Wang J, Liu W, Kang Y, Li Z, Hao C, Qi S: Exploring Gender Differences in the Association Between TyG Index and COPD: A Cross-Sectional Study from NHANES 1999-2018. *Int J Chron Obstruct Pulmon Dis* 2024, 19:2001-2010.

12. Shoelson SE, Lee J, Goldfine AB: Inflammation and insulin resistance. *J Clin Invest* 2006, 116:1793-1801.

13. Cardet JC, Ash S, Kusa T, Camargo CA, Jr., Israel E: Insulin resistance modifies the association between obesity and current asthma in adults. *Eur Respir J* 2016, 48:403-410.

14. Fermont JM, Masconi KL, Jensen MT, Ferrari R, Di Lorenzo VAP, Marott JM, Schuetz P, Watz H, Waschki B, Mullerova H, et al: Biomarkers and clinical outcomes in COPD: a systematic review and meta-analysis. *Thorax* 2019, 74:439-446.

15. Leuzzi G, Galeone C, Taverna F, Suatoni P, Morelli D, Pastorino U: C-reactive

protein level predicts mortality in COPD: a systematic review and meta-analysis. *Eur Respir Rev* 2017, 26.

16. Agassandian M, Shurin GV, Ma Y, Shurin MR: C-reactive protein and lung diseases. *Int J Biochem Cell Biol* 2014, 53:77-88.
17. Li J, Ye P, Peng X, Xiang G: The roles of lipids and inflammation in the association between the triglyceride-glucose index and arterial stiffness: evidence from two large population-based surveys. *Lipids Health Dis* 2024, 23:190.
18. Li T, Wang P, Wang X, Liu Z, Zhang Z, Zhang Y, Wang Z, Feng Y, Wang Q, Guo X, et al: Inflammation and Insulin Resistance in Diabetic Chronic Coronary Syndrome Patients. *Nutrients* 2023, 15.
19. Luo P, He J, Wan X, Li M, Zhu Z, Chen L, Hang D, Su J, Tao R, Zhou J, Fan X: Association between birth weight and chronic obstructive pulmonary disease in the UK Biobank: a prospective cohort study. *BMJ Open Respir Res* 2024, 11.
20. Zhu Z, Wan X, Liu J, Zhang D, Luo P, Du W, Chen L, Su J, Hang D, Zhou J, Fan X: Vitamin D status and chronic obstructive pulmonary disease risk: a prospective UK Biobank study. *BMJ Open Respir Res* 2023, 10.
21. Quanjer PH, Stanojevic S, Cole TJ, Baur X, Hall GL, Culver BH, Enright PL, Hankinson JL, Ip MS, Zheng J, et al: Multi-ethnic reference values for spirometry for the 3-95-yr age range: the global lung function 2012 equations. *Eur Respir J* 2012, 40:1324-1343.
22. Ramdas Nayak VK, Satheesh P, Shenoy MT, Kalra S: Triglyceride Glucose (TyG) Index: A surrogate biomarker of insulin resistance. *J Pak Med Assoc* 2022, 72:986-

988.

23. Zhan J, Wei Q, Guo W, Liu Z, Chen S, Huang Q, Liang S, Cai D: Evaluating the triglyceride glucose index as a predictive biomarker for osteoporosis in patients with type 2 diabetes. *Front Endocrinol (Lausanne)* 2025, 16:1534232.

24. Paudel S, Ahmadi M, Phongsavan P, Hamer M, Stamatakis E: Do associations of physical activity and sedentary behaviour with cardiovascular disease and mortality differ across socioeconomic groups? A prospective analysis of device-measured and self-reported UK Biobank data. *Br J Sports Med* 2023, 57:921-929.

25. Su J, Li M, Wan X, Yu H, Wan Y, Hang D, Lu Y, Tao R, Wu M, Zhou J, Fan X: Associations of diabetes, prediabetes and diabetes duration with the risk of chronic obstructive pulmonary disease: A prospective UK Biobank study. *Diabetes Obes Metab* 2023, 25:2575-2585.

26. Feng G, Yang M, Xu L, Liu Y, Yu J, Zang Y, Shen S, Zheng X: Combined effects of high sensitivity C-reactive protein and triglyceride-glucose index on risk of cardiovascular disease among middle-aged and older Chinese: Evidence from the China Health and Retirement Longitudinal Study. *Nutr Metab Cardiovasc Dis* 2023, 33:1245-1253.

27. Liu H, Wang L, Wang H, Hao X, Du Z, Li C, Hou X: Triglyceride-glucose index correlates with the incidences and prognoses of cardiac arrest following acute myocardial infarction: data from two large-scale cohorts. *Cardiovasc Diabetol* 2025, 24:108.

28. Zaigham S, Tanash H, Nilsson PM, Muhammad IF: Triglyceride-Glucose Index is

a Risk Marker of Incident COPD Events in Women. *Int J Chron Obstruct Pulmon Dis* 2022, 17:1393-1401.

29. Wiegman CH, Li F, Ryffel B, Togbe D, Chung KF: Oxidative Stress in Ozone-Induced Chronic Lung Inflammation and Emphysema: A Facet of Chronic Obstructive Pulmonary Disease. *Front Immunol* 2020, 11:1957.
30. Forno E, Han YY, Muzumdar RH, Celedon JC: Insulin resistance, metabolic syndrome, and lung function in US adolescents with and without asthma. *J Allergy Clin Immunol* 2015, 136:304-311 e308.
31. Ruan W, Deng J, Ying K: Novel Aspects of Insulin-like Growth Factor 1/insulin Network in Chronic Inflammatory Airway Disease. *Curr Med Chem* 2020, 27:7256-7263.
32. Ferreira SS, Oliveira MA, Tsujita M, Nunes FPB, Casagrande FB, Gomes E, Russo M, Tavares de Lima W, Martins JO: Insulin Modulates the Immune Cell Phenotype in Pulmonary Allergic Inflammation and Increases Pulmonary Resistance in Diabetic Mice. *Front Immunol* 2020, 11:84.
33. Kheradmand F, Zhang Y, Corry DB: Contribution of adaptive immunity to human COPD and experimental models of emphysema. *Physiol Rev* 2023, 103:1059-1093.
34. Yao X, Huang J, Zhong H, Shen N, Faggioni R, Fung M, Yao Y: Targeting interleukin-6 in inflammatory autoimmune diseases and cancers. *Pharmacol Ther* 2014, 141:125-139.
35. Michaeloudes C, Abubakar-Waziri H, Lakhdar R, Raby K, Dixey P, Adcock IM, Mumby S, Bhavsar PK, Chung KF: Molecular mechanisms of oxidative stress in

asthma. *Mol Aspects Med* 2022, 85:101026.

36. Cui C, Liu L, Qi Y, Han N, Xu H, Wang Z, Shang X, Han T, Zha Y, Wei X, Wu Z: Joint association of TyG index and high sensitivity C-reactive protein with cardiovascular disease: a national cohort study. *Cardiovasc Diabetol* 2024, 23:156.
37. Wu Z, Cheng C, Sun X, Wang J, Guo D, Chen S, Zhang Y, Liu X, Liu L, Zhang C, Yang J: The synergistic effect of the triglyceride-glucose index and serum uric acid on the prediction of major adverse cardiovascular events after coronary artery bypass grafting: a multicenter retrospective cohort study. *Cardiovasc Diabetol* 2023, 22:103.
38. Larsson SC, Burgess S: Appraising the causal role of smoking in multiple diseases: A systematic review and meta-analysis of Mendelian randomization studies. *EBioMedicine* 2022, 82:104154.
39. Castan-Abad MT, Montserrat-Capdevila J, Godoy P, Marsal JR, Ortega M, Alseda M, Barbe F: Diabetes as a risk factor for severe exacerbation and death in patients with COPD: a prospective cohort study. *Eur J Public Health* 2020, 30:822-827.
40. Chen L, Xiong H, Wen Q, Lv J, Sun D, Pei P, Yang L, Chen Y, Du H, Li L, et al: The Role of Active and Passive Smoking in Chronic Obstructive Pulmonary Disease and Systemic Inflammation: A 12-year Prospective Study in China. *J Epidemiol Glob Health* 2024, 14:1332-1340.
41. Zhou Y, Wang D, Liu S, Lu J, Zheng J, Zhong N, Ran P: The association between BMI and COPD: the results of two population-based studies in Guangzhou, China. *COPD* 2013, 10:567-572.

42. Li W, Liu T, Qian L, Wang Y, Ma X, Cao L, Zhang Q, Qu J: Insulin resistance and inflammation mediate the association of abdominal obesity with colorectal cancer risk. *Front Endocrinol (Lausanne)* 2022, 13:983160.

43. Chen C, Lu Z, Wang X, Zhang J, Zhang D, Li S: The chain mediating role of C-reactive protein and triglyceride-glucose index between lung function and cognitive function in a systemic low-grade inflammation state. *J Psychiatr Res* 2022, 155:380-386.

SPRINGER NATURE
Author Services

Editing Certificate

This document certifies that the manuscript

The joint effect of triglyceride-glucose index and C-reactive protein levels on the risk of chronic obstructive pulmonary disease: A prospective cohort study.

prepared by the authors

Jialiu He, Mengxia Li, Pengfei Luo, Zheng Zhu, Jian Su, Ran Tao, Jinyi Zhou, Ming Wu, Xikang Fan

was edited for proper English language, grammar, punctuation, spelling, and overall style by one or more of the highly qualified English speaking editors at SNAS.

This certificate was issued on **July 2, 2025** and may be verified on the SNAS website using the verification code **1DCB-BD22-1A5F-6215-6972** .

Neither the research content nor the authors' intentions were altered in any way during the editing process. Documents receiving this certification should be English-ready for publication; however, the author has the ability to accept or reject our suggestions and changes. To verify the final SNAS edited version, please visit our verification page at secure.authorservices.springernature.com/certificate/verify.
If you have any questions or concerns about this edited document, please contact SNAS at support@as.springernature.com.

SNAS provides a range of editing, translation, and manuscript services for researchers and publishers around the world.
For more information about our company, services, and partner discounts, please visit authorservices.springernature.com.
